# A Second Pathogenic Protein, PolyGN2C‐iso2, Reveals a Dual‐Protein Pathology in Neuronal Intranuclear Inclusion Disease

**DOI:** 10.1002/advs.76702

**Published:** 2026-07-23

**Authors:** Kang Zhang, Wenhao Ma, Yi Zhou, Zhijie Wu, Pan Gao, Hongze Niu, Hongfei Tai, Tianyi Zhao, Zheyue Dong, Li Li, Yan Zhang, An Wang, Si Shen, Yueyang Li, Sifei Yu, Yan Peng, Wang Sheng, Xiaoyan Dong, Hua Pan, Kaibin Shi, Magdalena J. Koziol, Xiaobing Wu, Zaiqiang Zhang

**Affiliations:** ^1^ Department of Neurology Beijing Tiantan Hospital Capital Medical University Beijing China; ^2^ GeneCradle Therapeutics lnc. Beijing China; ^3^ Beijing Ruicy Institute of Gene Therapy for Rare Disease Beijing China; ^4^ Beijing University of Technology Beijing China; ^5^ Beijing Institute for Brain Research Chinese Academy of Medical Sciences & Peking Union Medical College Beijing China; ^6^ Chinese Institute for Brain Research (CIBR) Beijing China; ^7^ Research Unit of Medical Neurobiology Chinese Academy of Medical Sciences Beijing China; ^8^ Institute for Immunology Chinese Institutes for Medical Research (CIMR) Beijing China

**Keywords:** dual‐proteinopathy, GGC repeat expansion, mitochondrial dysfunction, neuronal intranuclear inclusion disease, NOTCH2NLC, PolyGN2C‐iso2

## Abstract

**Background:**

Neuronal intranuclear inclusion disease (NIID) pathogenesis has been strongly linked to uN2CpolyG translated from NOTCH2NLC transcript variant 1. However, emerging evidence suggests that NOTCH2NLC transcript variant 2 may also generate a disease‐relevant protein, PolyGN2C‐iso2, but isoform‐discriminating and antibody‐independent evidence remains incomplete.

**Methods:**

We characterize the aggregation propensity of PolyGN2C‐iso2 in vitro, develop isoform‐discriminating monoclonal antibodies for its detection in patient tissues, and perform targeted proteomic analysis of laser‐microdissected p62‐positive lesion cells. An AAV‐mediated mouse model expressing PolyG(108×)N2C‐iso2 is generated to assess its pathogenic potential, followed by behavioral, imaging, histopathological, proteomic, and functional analyses.

**Results:**

NOTCH2NLC transcript variant 2 generates a distinct protein, PolyGN2C‐iso2, which forms aggregates in vitro. Using developed monoclonal antibodies together with targeted proteomics, we provide evidence that PolyGN2C‐iso2 is present within the pathognomonic intranuclear inclusions in NIID patient tissues, where it co‐localizes with uN2CpolyG. The PolyG(108×)N2C‐iso2 mouse model recapitulates key pathological hallmarks of NIID, including white matter abnormalities and cognitive deficits not fully captured by previous models. Mechanistically, PolyGN2C‐iso2 expression is found to induce profound mitochondrial dysfunction.

**Conclusions:**

Our findings support the possibility that NIID involves a dual‐protein pathogenic process involving both uN2CpolyG and PolyGN2C‐iso2, which may have implications for therapeutic strategies targeting NOTCH2NLC‐derived pathogenic proteins.

## Graphical abstract

This study reveals that NOTCH2NLC transcript variant 2 generates PolyGN2C‐iso2, an aggregating protein present within intranuclear inclusions of NIID patient tissues. A novel mouse model expressing PolyGN2C‐iso2 recapitulates white matter abnormalities and cognitive deficits, mechanistically linked to mitochondrial dysfunction. These findings support a dual‐protein pathogenic process in NIID.

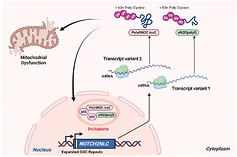

## 1 Introduction

Nucleotide repeat expansion disorders are a group of inherited diseases caused by abnormal expansions of short tandem repeats in the genome and represent an important genetic mechanism underlying neurodegenerative and neuromuscular diseases [1, 2, 3, 4]. More than 50 neurological disorders have been linked to repeat expansions, among which CGG/GGC repeat expansion diseases have recently attracted increasing attention [5, 6, 7]. Neuronal intranuclear inclusion disease (NIID) and oculopharyngodistal myopathy type 3 (OPDM3), two rare disorders with overlapping neurodegenerative and neuromuscular manifestations, have been genetically linked to GGC/CGG repeat expansions in *NOTCH2NLC* [8, 9, 10, 11]. Recent studies demonstrated that the expanded repeats can be translated through a previously unrecognized upstream open reading frame, generating aggregation‐prone polyglycine‐containing proteins [7, 12, 13]. These findings led to the emerging concept of “polyG diseases,” in which repeat‐derived polyG proteins may act as shared pathogenic drivers and contribute to p62‐positive intranuclear inclusion formation [7, 12, 13]. More recent work has further shown that polyG‐dependent toxicity can involve downstream mechanisms such as microglia‐mediated neurodegeneration, mitochondrial and white‐matter pathology, and sequestration of endogenous glycine‐rich proteins such as FAM98B, leading to disrupted tRNA processing [14, 15]. However, the molecular mechanisms underlying NOTCH2NLC‐related polyG diseases remain incompletely understood.

Among these diseases, NIID provides a particularly important model for understanding NOTCH2NLC‐related polyG pathology, as it is a multisystem neurodegenerative disorder characterized by widespread eosinophilic intranuclear inclusions in the central nervous system and multiple visceral organs [5, 16]. This multisystem inclusion pathology has facilitated tissue‐based diagnosis and shaped subsequent genetic investigations of NIID. Major diagnostic advances include the introduction of skin biopsy in 2011 [17] and the seminal 2019 discovery of GGC repeat expansions in *NOTCH2NLC* [8] as the primary genetic cause in East Asian populations, with repeat counts >60 considered pathogenic [9, 18]. Despite these advances, no established disease‐modifying therapy is currently available for NIID, highlighting an urgent need to fully understand the molecular pathogenesis [5, 19]. The current pathogenic model is centered on *NOTCH2NLC* transcript variant 1, where a 5' UTR GGC expansion drives the production of uN2CpolyG, a toxic polyglycine protein that forms the characteristic inclusions [12, 20]. Together, these studies have shaped a prevailing uN2CpolyG‐centered model of NIID pathogenesis, in which uN2CpolyG is considered the principal NOTCH2NLC‐derived toxic polyglycine protein driving intranuclear inclusion formation and neurodegeneration [12, 14].

However, several observations suggest that the current uN2CpolyG‐centered model may not fully explain the protein composition of NIID inclusions. Immunofluorescence analysis of NIID brain tissue has shown different inclusion detection rates between antibodies: the 4D12 antibody labeled 89.5% of inclusions, whereas the uN2CpolyG‐specific antibody 4C4 detected 56.9% [12, 21]. This difference raises the possibility that additional *NOTCH2NLC*‐derived protein species may also be present in NIID inclusions. In this context, NOTCH2NLC transcript variant 2 is of particular interest, because the GGC repeat is located within its coding sequence and may therefore be translated into a distinct polyglycine‐containing chimeric protein, herein designated PolyGN2C‐iso2 [22, 23]. Previous studies have implicated protein products derived from transcript variant 2 in NIID‐related pathology and toxicity [22, 23]. In particular, Wang et al. reported p62‐positive intranuclear immunoreactivity in NIID skin samples using a commercially available antibody annotated to recognize NOTCH2NLC isoform 2 [23]. However, given the high sequence homology among human‐specific NOTCH2NL paralogues [24, 25], prior antibody‐based evidence did not fully resolve whether endogenous PolyGN2C‐iso2 itself is an isoform‐specific component of NIID inclusions. Therefore, isoform‐discriminating and antibody‐independent evidence remained necessary to define the endogenous contribution of PolyGN2C‐iso2 to NIID inclusions, its relationship with uN2CpolyG, and its autonomous pathogenic potential in vivo.

In this study, we addressed these issues by developing PolyGN2C‐iso2‐specific antibodies, performing targeted proteomic analysis to detect PolyGN2C‐iso2‐discriminating peptide evidence, and generating an AAV‐based mouse model expressing PolyG(108×)N2C‐iso2. We sought to clarify the inclusion composition of PolyGN2C‐iso2 using isoform‐discriminating and antibody‐independent approaches and to assess its independent pathogenic potential in vivo. Our findings provide evidence that PolyGN2C‐iso2 contributes to NIID pathology and support an expanded disease model in which both uN2CpolyG and PolyGN2C‐iso2 are involved. This framework may have implications for future therapeutic strategies targeting NOTCH2NLC‐derived pathogenic proteins.

## 2 Results

### 2.1 Pathogenic GGC Repeats Drive PolyGN2C‐iso2 Aggregation and Co‐Localization With uN2CpolyG In Vitro

To investigate the aggregation properties of NOTCH2NLC transcript variant 2 (TV2)‐derived PolyGN2C‐iso2, we characterized this protein product in cultured cells. TV2 contains the GGC repeat within its coding sequence, predicting the translation of a distinct chimeric protein (Figure 1A). Computational modeling predicted that the pathogenic polyglycine expansion induces a substantial conformational change, suggesting a high propensity for aggregation (Figure 1B). We constructed expression plasmids for PolyGN2C‐iso2 with either 14 (wild‐type) or 64 (pathogenic) GGC repeats (Figure 1C and Figure S1A,B). Western blot analysis of transfected HEK293T and U2OS cells confirmed the expression of both wild‐type and pathogenic proteins, with the latter migrating at a higher molecular weight as expected (Figure 1D,E). To further verify the identity of the translated TV2‐derived product, we performed in‐gel LC‐MS/MS analysis using the gel band excised from U2OS cells expressing PolyG(64×)N2C‐iso2‐Flag. Multiple peptides mapping to the predicted PolyGN2C‐iso2‐3×Flag protein product were identified, including peptides from the polyG‐containing N‐terminal region, internal PolyGN2C‐iso2 sequences, and C‐terminal/FLAG‐junction regions (Figure S1C). These data support that the TV2‐PolyG‐Flag construct produces the expected PolyGN2C‐iso2 translation product. Importantly, anti‐FLAG immunoblotting further revealed high‐molecular‐weight PolyGN2C‐iso2 species above 250 kDa in cells expressing pathogenic PolyG(64×)N2C‐iso2, whereas these signals were absent in both vector‐transfected cells and cells expressing wild‐type PolyG(14×)N2C‐iso2 (Figure 1D,E). These data support the formation of high‐molecular‐weight aggregate species by pathogenic PolyGN2C‐iso2. Immunofluorescence microscopy revealed that while wild‐type PolyG(14×)N2C‐iso2 showed a diffuse distribution, the pathogenic PolyG(64×)N2C‐iso2 formed numerous distinct aggregates in both the cytoplasm and nucleus (Figure 1F,G). Quantitative analysis indicated that these aggregates were predominantly located in the cytoplasm (Figure 1H,I). These PolyG(64×)N2C‐iso2 inclusions were pathologically relevant, as demonstrated by their robust co‐localization with the autophagy adapter protein p62 (Figure S1D–F). Given that both transcript variants coexist in NIID patients, we next investigated the potential for co‐assembly of their protein products. Using a bicistronic vector to co‐express HA‐tagged uN2CpolyG and FLAG‐tagged PolyGN2C‐iso2, we observed extensive co‐localization of both proteins within the same inclusions (Figure 1J,K). Quantitative analysis further confirmed a high degree of co‐localization between HA‐positive uN2CpolyG and FLAG‐positive PolyGN2C‐iso2 in both intranuclear and intracytoplasmic inclusions (Figure 1J,K). Expression from this construct was verified by Western blot (Figure S1G).

**FIGURE 1:**
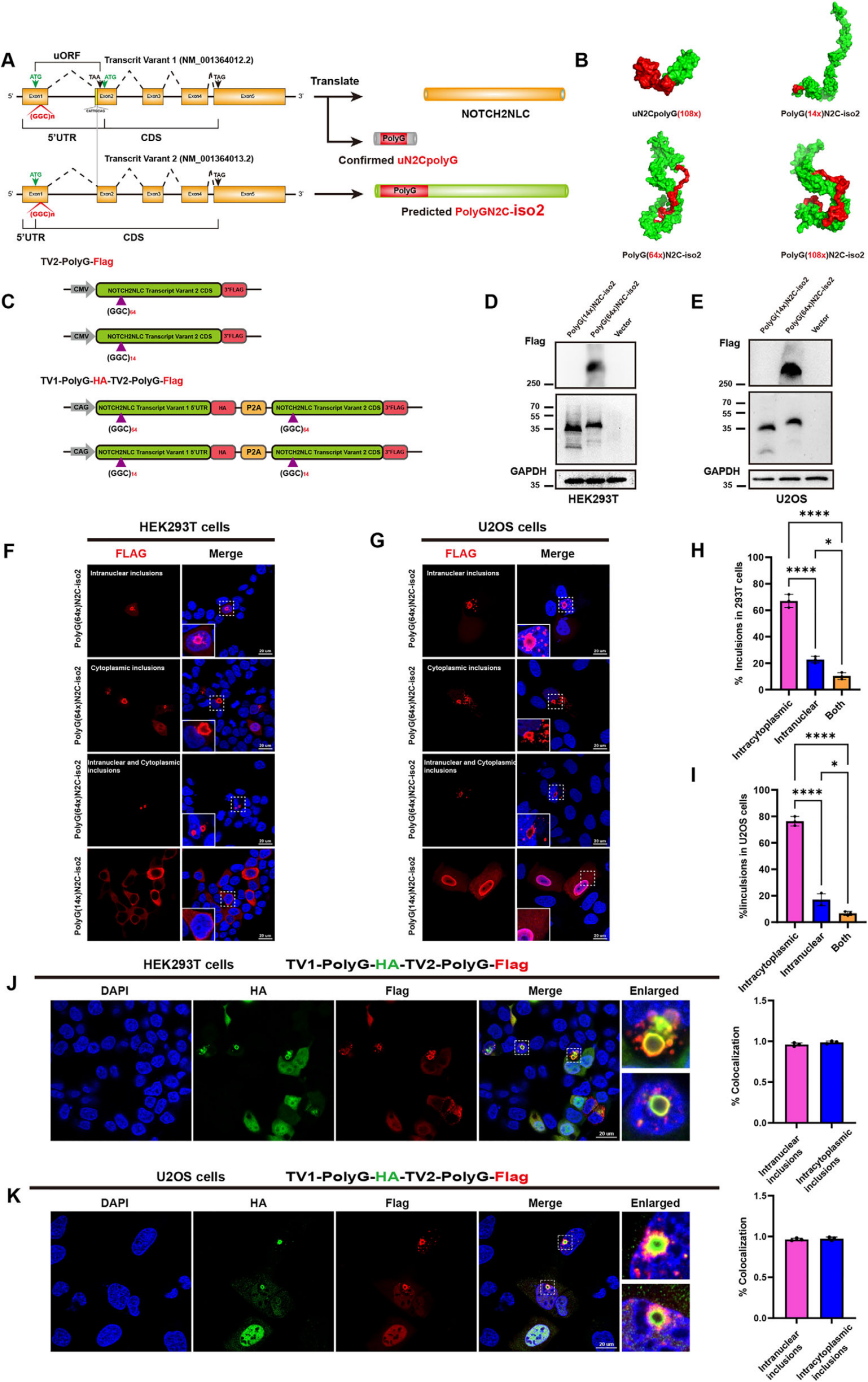
GGC repeat expansion in *NOTCH2NLC* transcript variant 2 generates PolyGN2C‐iso2 inclusions. (A) Schematic of *NOTCH2NLC* transcript variants 1 (TV1) and 2 (TV2). The GGC repeat (red) is in the 5' UTR of TV1, producing uN2CpolyG, and in the coding sequence (CDS) of TV2, predicting to generate the PolyGN2C‐iso2 fusion protein. (B) Predicted 3D structures of uN2CpolyG and PolyGN2C‐iso2 with different GGC repeat lengths (14×, 64×, 108×) using AlphaFold3. Models are shown as molecular surfaces with the PolyG domain in red and the main protein in green. (C) Schematic of expression constructs used. TV2‐PolyG‐Flag contains the TV2 CDS with a C‐terminal 3×Flag tag. TV1‐PolyG‐HA‐TV2‐PolyG‐Flag combines the TV1 5' UTR (generating HA‐tagged uN2CpolyG) and the TV2 CDS (generating Flag‐tagged PolyGN2C‐iso2). Constructs with 14× or 64× GGC repeats were generated. See Figure S1 for plasmid maps and sequences. (D,E) Immunoblots of lysates from HEK293T (D) and U2OS (E) cells transfected with TV2‐PolyG‐Flag constructs (14× or 64× GGC repeats) or vector control. Blots were probed with an anti‐FLAG antibody 48 h post‐transfection. The upper panels show high‐molecular‐weight PolyGN2C‐iso2 aggregate species above 250 kDa, while the lower panels show monomeric PolyGN2C‐iso2. GAPDH served as a loading control. (F and G) Representative immunofluorescence images of HEK293T (F) and U2OS (G) cells transfected with TV2‐PolyG‐Flag (14× or 64× repeats). Cells were stained for FLAG (green) and with DAPI (blue). Scale bars, 10 µm. (H and I) Quantification of the percentage of transfected cells showing cytoplasmic or nuclear inclusions in HEK293T (H) and U2OS (I) cells from (F) and (G). (J,K) Representative immunofluorescence images and quantification showing colocalization of uN2CpolyG (HA, red) and PolyG(64×)N2C‐iso2 (FLAG, green) in HEK293T (J) and U2OS (K) cells transfected with the TV1‐PolyG‐HA‐TV2‐PolyG‐Flag (64×) construct. Nuclei are stained with DAPI (blue). Scale bars, 10 µm. For (H and I), data are from three independent experiments (*n* = 3), with at least 100 cells counted per experiment. Data are presented as mean ± SEM. Statistical significance for differences among cytoplasmic, nuclear, and dual‐localized inclusions was determined by one‐way ANOVA with Tukey's post‐hoc test. ^*^ *P* < 0.05, ^****^ *P* < 0.0001.

Collectively, these in vitro findings show that PolyGN2C‐iso2 is an aggregation‐prone protein whose pathogenic form readily forms p62‐positive inclusions and co‐assembles with uN2CpolyG, supporting a dual‐protein aggregation model for NIID.

### 2.2 PolyGN2C‐iso2 is a Component of Intranuclear Inclusions in NIID Patients

Having demonstrated co‐aggregation in vitro, we next sought to determine whether PolyGN2C‐iso2 is present in the pathognomonic inclusions of NIID patients using isoform‐discriminating reagents. We analyzed skin biopsies from three genetically confirmed NIID patients who exhibited typical clinical and neuroimaging features of the disease (Figure 2A and Figure S2A,B and Table S1). To achieve specific detection, we developed two new monoclonal antibodies, M11F6 and M38D5, targeting distinct epitopes of PolyGN2C‐iso2 (Figure 2B and Figure S2C,D). M11F6 recognizes a unique C‐terminal sequence, while M38D5 targets an epitope adjacent to the polyG tract. The specificity of these antibodies was validated in transfected U2OS cells (Figure S2E). In addition, Western blotting with M38D5 and M11F6 detected high‐molecular‐weight PolyG(64×)N2C‐iso2 species above 250 kDa, further confirming that both antibodies recognize aggregated PolyGN2C‐iso2 species biochemically (Figure S2F).

**FIGURE 2:**
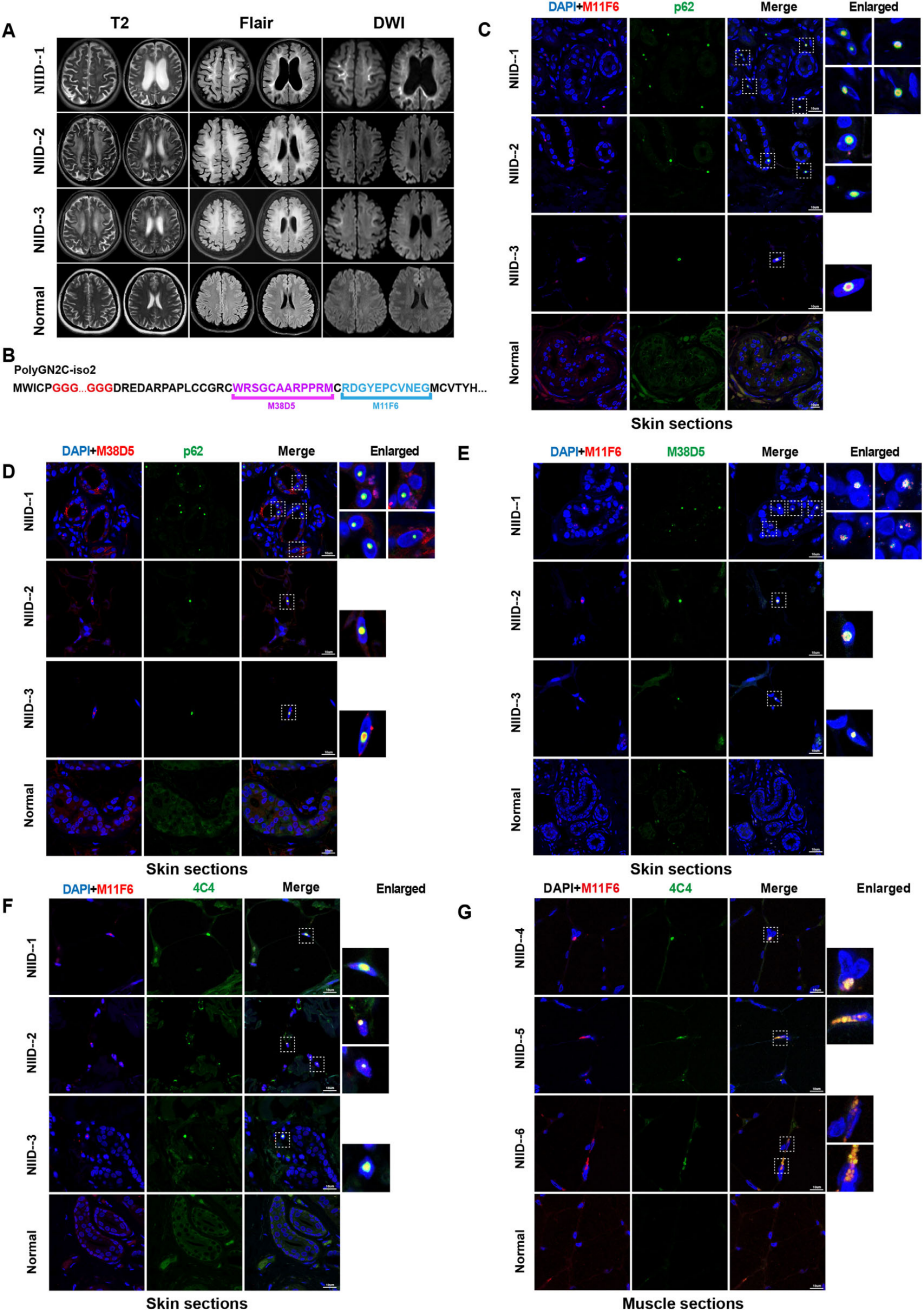
Clinical MRI features and detection of PolyGN2C‐iso2‐positive intranuclear inclusions in NIID patient tissues. (A) Representative brain MRI images from NIID patients (NIID‐1 to NIID‐3) and a healthy control. T2‐weighted, T2‐FLAIR, and diffusion‐weighted imaging (DWI) show characteristic high‐intensity signals in the cerebral white matter and along the corticomedullary junction in NIID patients. (B) Schematic diagram of the amino acid sequences of the PolyGN2C‐iso2 protein targeted by the mouse monoclonal antibodies M38D5 and M11F6. (C to F) Representative immunofluorescence confocal images of skin biopsy sections from NIID patients (NIID‐1 to NIID‐3) and a healthy control. (C) Double staining for PolyGN2C‐iso2 (M11F6, red) and the inclusion marker p62 (green). (D) Double staining for PolyGN2C‐iso2 (M38D5, red) and p62 (green). (E) Double staining was performed using directly conjugated M11F6–Alexa Fluor 555 (red) and M38D5–Alexa Fluor 488 (green). (F) Double staining for PolyGN2C‐iso2 (M11F6, red) and uN2CpolyG (4C4, green). (G) Representative immunofluorescence confocal images of skeletal muscle sections from NIID patients (NIID‐4 to NIID‐6) and a healthy control, showing double staining for PolyGN2C‐iso2 (M11F6, red) and uN2CpolyG (4C4, green).

Immunohistochemical analysis of patient skin sections using the M11F6 antibody revealed that p62‐positive inclusions within sweat gland cells, fibroblasts, and adipocytes were also positive for PolyGN2C‐iso2 (Figure 2C). These inclusions were similarly labeled by M38D5, confirming they contained a polyG‐expanded protein (Figure 2D). The extensive co‐localization of signals from M11F6 and M38D5 within the same nuclear aggregates strongly supported the specific detection of PolyGN2C‐iso2 (Figure 2E). To define the relationship between the two pathogenic proteins in situ, we co‐stained sections with M11F6 and the uN2CpolyG‐specific antibody 4C4 [3, 8, 17]. This analysis demonstrated robust co‐localization of PolyGN2C‐iso2 and uN2CpolyG within the same nuclear inclusions (Figure 2F). Subsequently, we performed quantitative analysis of PolyGN2C‐iso2‐positive intranuclear inclusions in patient skin tissues. In skin biopsy specimens from NIID‐1 to NIID‐3, at least 30 p62‐positive intranuclear inclusions were counted per patient. M11F6‐positive signals were detected in 89.0 ± 2.5% of p62‐positive intranuclear inclusions, indicating that PolyGN2C‐iso2 is present in the majority of p62‐positive pathological inclusions (Table 1). To define the relationship between PolyGN2C‐iso2 and uN2CpolyG, we quantified 4C4 positivity among M11F6‐positive intranuclear inclusions. Among M11F6‐positive inclusions, 86.7 ± 3.6% were also positive for 4C4, demonstrating extensive co‐localization between PolyGN2C‐iso2 and NOTCH2NLC transcript variant 1‐derived uN2CpolyG in patient skin tissues (Table 1).

**TABLE 1:** Quantification of PolyGN2C‐iso2‐ and uN2CpolyG‐positive intranuclear inclusions in NIID patient tissues.

Tissue	Patient	Quantification	Patient‐level counts	Mean±SEM
Skin	NIID‐1	M11F6+ among p62+ inclusions	31/33	89.0 ± 2.5%
	NIID‐2	M11F6+ among p62+ inclusions	32/37	
	NIID‐3	M11F6+ among p62+ inclusions	26/30	
Skin	NIID‐1	4C4+ among M11F6+ inclusions	21/26	86.7 ± 3.6%
	NIID‐2	4C4+ among M11F6+ inclusions	27/29	
	NIID‐3	4C4+ among M11F6+ inclusions	19/22	
Muscle	NIID‐4	4C4+ among M11F6+ inclusions	21/24	84.8 ± 4.3%
	NIID‐5	4C4+ among M11F6+ inclusions	13/17	
	NIID‐6	4C4+ among M11F6+ inclusions	19/21	

Data are presented as mean ± SEM, with each patient considered as one biological replicate. In skin biopsies, at least 30 p62‐positive intranuclear inclusions were counted per patient for the M11F6/p62 analysis, and at least 22 M11F6‐positive intranuclear inclusions were counted per patient for the 4C4/M11F6 analysis. In skeletal muscle biopsies, at least 17 M11F6‐positive intranuclear inclusions were counted per patient.

To further assess whether PolyGN2C‐iso2 is also present in other disease‐relevant affected tissues, we analyzed skeletal muscle biopsy specimens from an independent group of NIID patients, NIID‐4 to NIID‐6. Double immunofluorescence staining with M11F6 and 4C4 revealed intranuclear inclusion‐like signals that were double‐positive for PolyGN2C‐iso2 and uN2CpolyG in skeletal muscle tissues (Figure 2G). Quantitative analysis showed that 84.8 ± 4.3% of M11F6‐positive intranuclear inclusions were also positive for 4C4, with at least 17 M11F6‐positive inclusions counted per patient (Table 1). These findings indicate that the coexistence of PolyGN2C‐iso2 and uN2CpolyG is not restricted to skin biopsy specimens but is also observed in skeletal muscle tissue from NIID patients.

Together, these antibody‐based analyses provide isoform‐discriminating patient‐tissue evidence that PolyGN2C‐iso2 is a component of NIID intranuclear inclusions and extensively co‐localizes with uN2CpolyG in both skin and skeletal muscle.

### 2.3 Targeted Proteomics Analysis Detects PolyGN2C‐iso2 in p62‐Positive NIID Lesion Cells

To further substantiate the immunostaining evidence at the protein level, we analyzed mass spectrometry datasets generated from laser‐microdissected p62‐positive sweat gland cells from skin biopsy specimens of 20 genetically confirmed patients with NIID, followed by Skyline‐based targeted proteomic analysis (Figure 3A). Given the high sequence similarity between NOTCH2NLC and closely related NOTCH2NL paralogues, we first performed sequence alignment of PolyGN2C‐iso2, uN2CpolyG, NOTCH2NLC isoforms, and NOTCH2NL paralogue‐derived sequences. This analysis identified N‐terminal peptide regions capable of discriminating PolyGN2C‐iso2 from uN2CpolyG and other closely related NOTCH2NL‐derived sequences (Figure 3B).

**FIGURE 3:**
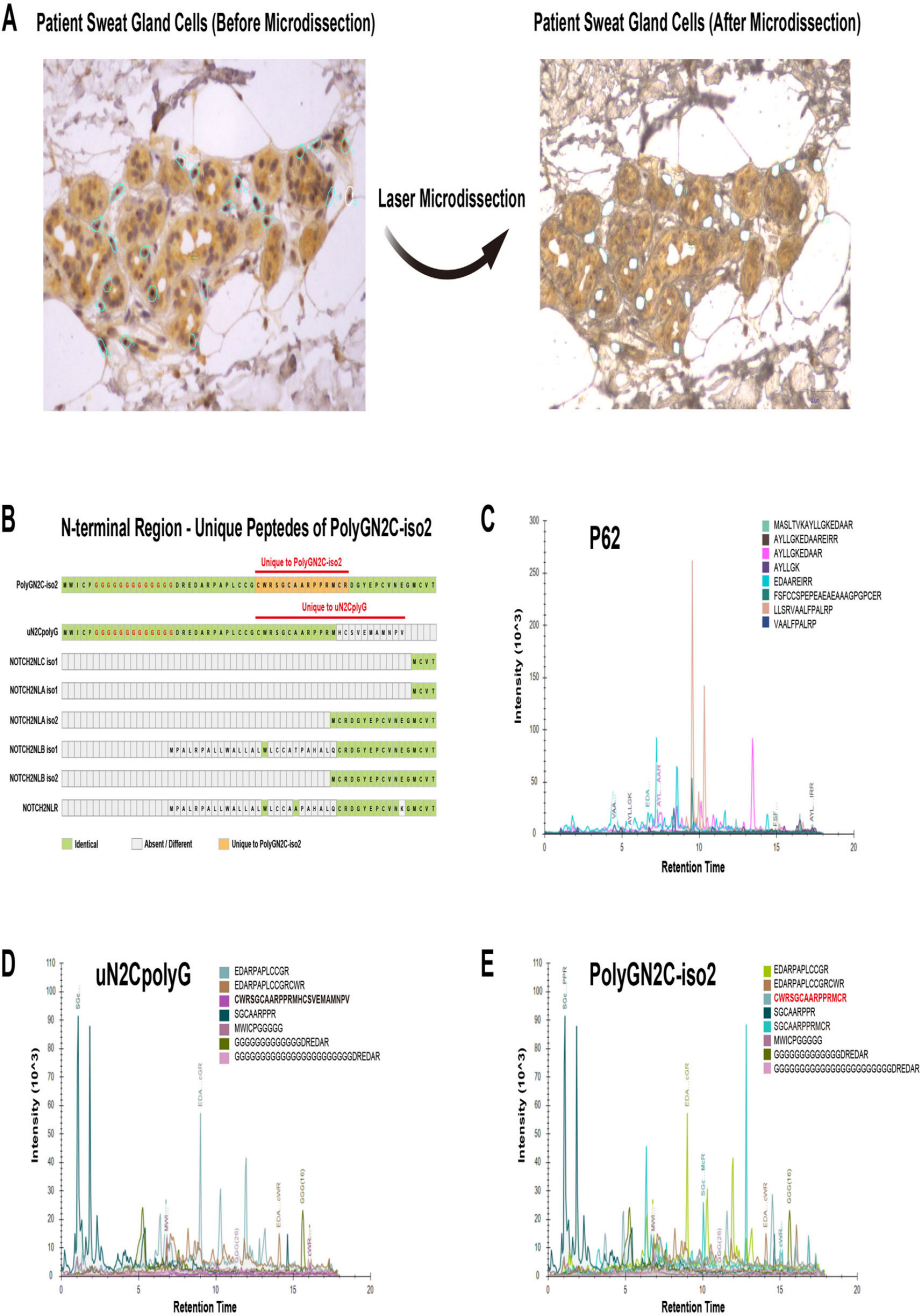
Targeted proteomic analysis detects PolyGN2C‐iso2 in laser‐microdissected p62‐positive sweat gland lesion cells from NIID patients. (A) Representative images of p62 immunohistochemical staining in skin tissue from an NIID patient before and after laser microdissection. Laser microdissection was used to isolate p62‐positive sweat gland cells harboring intranuclear inclusions for proteomic analysis. (B) N‐terminal sequence comparison highlighting peptide regions that distinguish PolyGN2C‐iso2 from uN2CpolyG and related NOTCH2NL paralogue‐derived sequences. Red lines indicate isoform‐discriminating surrogate peptide regions used for targeted Skyline extraction. (C) Representative Skyline‐extracted chromatograms of p62‐derived peptides from laser‐microdissected p62‐positive sweat gland cells of NIID patients. (D) Representative Skyline‐extracted chromatograms of uN2CpolyG‐associated peptides. (E) Representative Skyline‐extracted chromatograms of PolyGN2C‐iso2‐associated peptides. The selected PolyGN2C‐iso2‐discriminating surrogate peptide CWRSGCAARPPRMCR is highlighted in red.

Consistent with the laser microdissection of p62‐positive lesion cells, multiple p62‐derived peptides were detected in the same patient datasets (Figure 3C). We then used Skyline to extract representative peptide signals corresponding to uN2CpolyG and PolyGN2C‐iso2. The uN2CpolyG‐associated surrogate peptide CWRSGCAARPPRMHCSVEMAMNPV was detected in NIID patient samples (Figure 3D). Importantly, the PolyGN2C‐iso2‐discriminating surrogate peptide CWRSGCAARPPRMCR was also detected in the same patient‐derived p62‐positive lesion cell datasets (Figure 3E). These results provide antibody‐independent protein‐level evidence that PolyGN2C‐iso2 is present in NIID pathological lesion cells.

We next performed semi‐quantitative analysis of the extracted total peak areas of representative uN2CpolyG‐ and PolyGN2C‐iso2‐associated surrogate peptides across patient samples. The uN2CpolyG/PolyGN2C‐iso2 surrogate‐peptide signal ratio was below 1 in most analyzed NIID samples (Figure S3A,B). Based on the summed total peak areas of the selected representative peptides, PolyGN2C‐iso2 accounted for approximately 79% of the combined NOTCH2NLC‐derived surrogate‐peptide signal, whereas uN2CpolyG accounted for approximately 21% (Figure S3C). Because these estimates were derived from distinct surrogate peptides without isotope‐labeled internal standards, they should be interpreted as semi‐quantitative relative surrogate‐peptide signals rather than absolute protein abundance or copy‐number ratios. Nevertheless, these targeted proteomic data further indicate that PolyGN2C‐iso2 is a readily detectable NOTCH2NLC‐derived pathogenic protein species in p62‐positive NIID lesion cells.

### 2.4 Expression of PolyG(108×)N2C‐iso2 is Sufficient to Recapitulate NIID‐Like Neuropathology in Mice

Although patient‐tissue data supported the presence of PolyGN2C‐iso2 in inclusions, the autonomous pathogenic capacity of PolyGN2C‐iso2 remained to be established. To address this, we generated a novel AAV‐mediated mouse model exclusively expressing pathogenic PolyG(108×)N2C‐iso2 via a single neonatal intracerebroventricular (ICV) injection (Figure 4A). After 14 weeks, mice expressing PolyG(108×)N2C‐iso2, but not control mice, developed widespread p62‐positive intranuclear inclusions throughout the central nervous system (CNS) and in peripheral organs (Figure 4B,C and Figure S4A). Ultrastructural analysis by electron microscopy revealed amorphous, non‐membrane‐bound inclusions within the nucleus and the presence of cytoplasmic autophagic vacuoles, suggesting disrupted autophagy (Figure 4D). Luxol Fast Blue staining confirmed significant demyelination, and cerebral MRI revealed ventricular enlargement and white matter signal abnormalities (Figure 4E,F). Immunofluorescence analysis confirmed that these inclusions contained PolyGN2C‐iso2 but were completely devoid of the uN2CpolyG protein (4C4‐negative), providing strong evidence that PolyGN2C‐iso2 alone is sufficient to drive inclusion formation in this model (Figure 4G,H and Figure S4B). PolyG(108×)N2C‐iso2 mice also exhibited robust astrogliosis (Figure S4C), further supporting the presence of NIID‐like neuropathological changes. Consistent with the autophagy‐related abnormalities observed in vivo, PolyG(64×)N2C‐iso2 expression in vitro led to the accumulation of both p62 and LC3‐II, indicative of impaired autophagic flux (Figure S4D).

**FIGURE 4:**
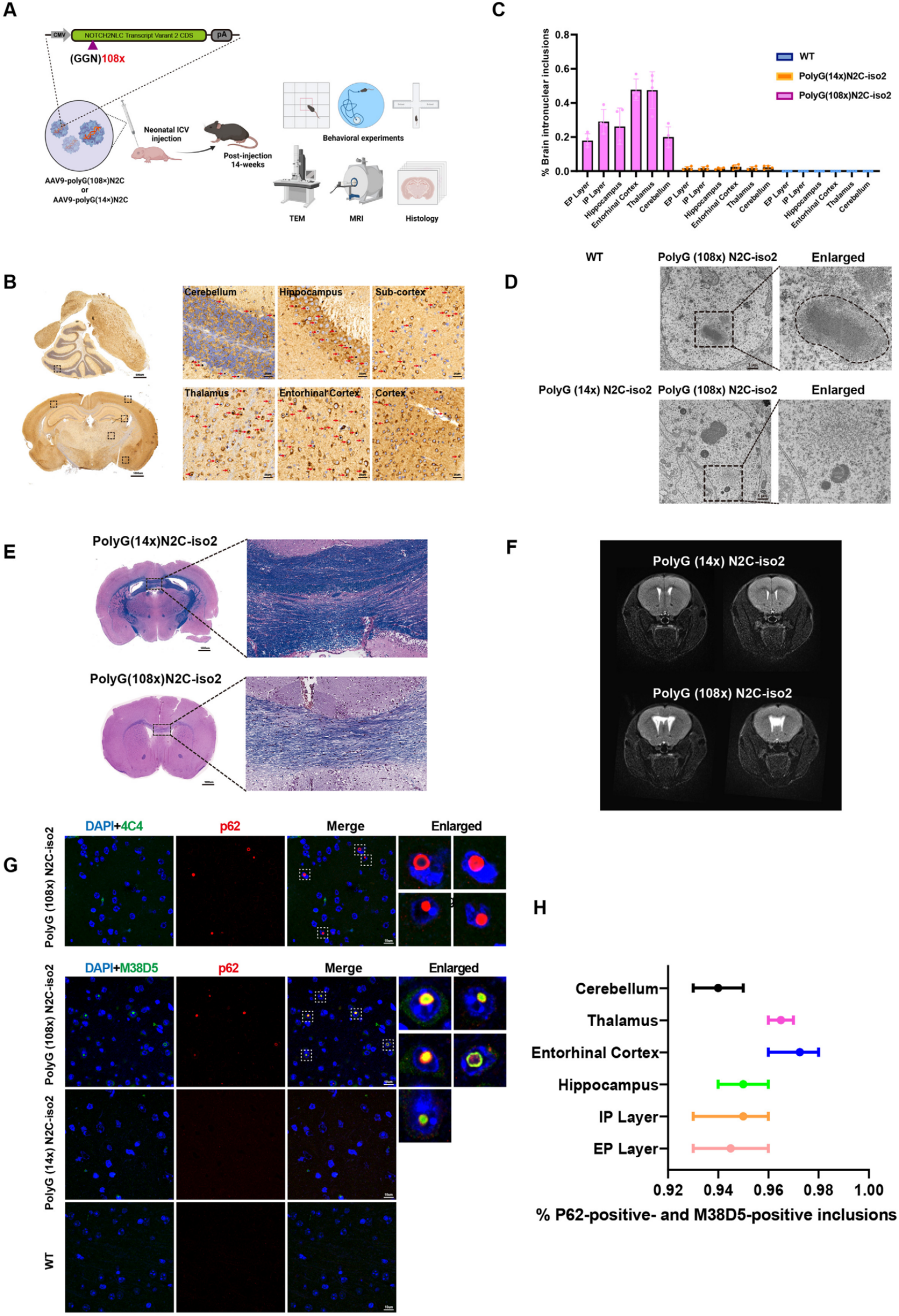
Expression of PolyG(108×)N2C‐iso2 induces NIID‐like pathology in a mouse model. (A) Schematic of the PolyG(108×)N2C‐iso2 mouse model and the experimental timeline for behavioral, imaging, and histopathological analyses. (B) Representative immunohistochemistry (IHC) for p62 in various brain regions of a PolyG(108×)N2C‐iso2 mouse. Red arrows indicate p62‐positive intranuclear inclusions. Scale bars, 500 to 1000 µm (left panels) and 25 µm (right panels). (C) Quantification of the percentage of cells with p62‐positive inclusions in different brain regions of PolyG(108×)N2C‐iso2, PolyG(14×)N2C‐iso2, and wild‐type (WT) mice. (D) Transmission electron microscopy (TEM) of an intranuclear inclusion in the brain of a PolyG(108×)N2C‐iso2 mouse. The inclusion consists of a high‐electron‐density core surrounded by fibrillar structures, with some being sequestered by autophagic vesicles. Scale bar, 1 µm. (E) Fast Blue staining of brain sections from PolyG(108×)N2C‐iso2 and PolyG(14×)N2C‐iso2 mice, showing demyelination (pale areas) in the white matter of the model mouse. Scale bar, 1000 µm. (F) Representative T2‐weighted brain MRI of PolyG(108×)N2C‐iso2 and PolyG(14×)N2C‐iso2 mice. The model mouse exhibits ventricular enlargement and white matter atrophy. (G) Representative immunofluorescence images of a brain section from a PolyG(108×)N2C‐iso2 mouse. Left panel shows colocalization of p62 (red) and M38D5 (green). Right panel shows an adjacent section stained for p62 (red) and 4C4 (green), demonstrating the absence of uN2CpolyG in the inclusions. Scale bar, 10 µm. (H) Quantification of the percentage of p62‐positive inclusions that are also positive for M38D5 in different brain regions of PolyG(108×)N2C‐iso2 mice. Data are presented as mean ± SEM; *n* = 4 mice per group. For (C), statistical significance was determined by two‐way ANOVA followed by Tukey's multiple comparisons test. For (H), data represent descriptive statistics.

Together, these results indicate that the exclusive expression of PolyGN2C‐iso2 is sufficient to induce the core NIID‐like neuropathological hallmarks in this model, thereby supporting its independent pathogenic role. This model was designed to test the autonomous pathogenic capacity of PolyGN2C‐iso2 rather than to quantitatively compare its toxicity with that of uN2CpolyG.

### 2.5 PolyG(108x)N2C‐iso2 Mice Recapitulate the Cognitive and Neuropsychiatric Deficits of NIID

To assess the functional consequences of PolyGN2C‐iso2 expression, we subjected the mice to a battery of behavioral tests at 14 weeks (Figure 5A). PolyG(108×)N2C‐iso2 mice exhibited severe phenotypes, including reduced survival and spontaneous seizure‐like episodes (Figure 5B and Movie S1), a clinical feature of NIID rarely captured in animal models. While body weights were comparable across groups (Figure 5C), the PolyG(108×)N2C‐iso2 mice showed profound cognitive impairment. In the Morris water maze, they displayed significantly longer escape latencies during training and spent less time in the target quadrant during the probe trial, indicating deficits in spatial learning and memory (Figure 5D–G).

**FIGURE 5:**
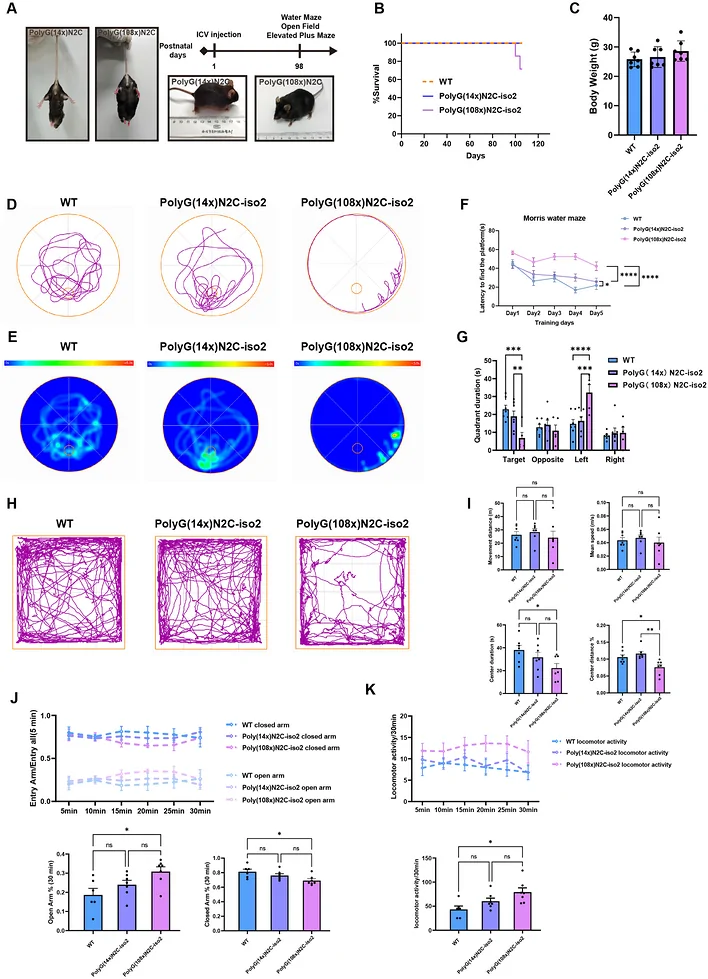
PolyG(108×)N2C‐iso2 expression induces NIID‐like behavioral deficits in mice. All behavioral tests were performed on male mice. (A) Schematic of the experimental timeline for lateral ventricle injection and behavioral testing. Representative images show PolyG(14×)N2C‐iso2 and PolyG(108×)N2C‐iso2 mice at 14 weeks of age. (B) Kaplan‐Meier survival analysis of WT, PolyG(14×)N2C‐iso2, and PolyG(108×)N2C‐iso2 mice. (C) Body weight of mice at 14 weeks of age. (D–G) Morris water maze test. (D) Representative swim tracks and (E) corresponding heatmaps from the probe trial. (F) Latency to find the hidden platform during the 5‐day training period. (G) Time spent in the target quadrant during the probe trial. (H,I) Open field test. (H) Representative locomotor tracks. (I) Quantification of total distance traveled, average speed, and time and distance in the center zone. (J,K) Elevated plus maze test. (J) Percentage of entries into the open arms, a measure of anxiety‐like behavior. (K) Total arm entries, a measure of general locomotor activity. Data are presented as mean ± SEM; *n* = 5–7 mice per group as indicated. Statistical significance was determined by two‐way repeated measures ANOVA with Tukey's multiple comparisons test. ^*^ *P* < 0.05, ^**^ *P* < 0.01, ^***^ *P* < 0.001.

Furthermore, the model recapitulated the complex neuropsychiatric profile of NIID. In the open field test, PolyG(108×)N2C‐iso2 mice did not show reduced total movement distance or mean speed but spent less time in the center, indicative of anxiety‐like behavior (Figure 5H,I). In the elevated plus maze, however, they paradoxically exhibited an increased percentage of entries into the open arms (Figure 5J,K). This behavioral signature is consistent with behavioral disinhibition resulting from frontal lobe dysfunction, a common finding in NIID patients. Although the open‐field and elevated plus maze assays are not dedicated motor‐function tests, PolyG(108×)N2C‐iso2 mice did not show reduced gross spontaneous locomotor activity at the 14‐week analysis time point. However, these data do not exclude skilled motor deficits, assay‐specific motor abnormalities, or later‐onset motor impairment as disease progresses.

Collectively, the PolyG(108×)N2C‐iso2 mouse model recapitulates several NIID‐like clinical features of NIID, including premature mortality, cognitive deficits, and a complex neuropsychiatric profile, providing a useful platform for investigating disease mechanisms.

### 2.6 Proteomic Analysis of NIID Patients Reveals Mitochondrial Metabolic Dysregulation

Having used targeted proteomics to verify the presence of PolyGN2C‐iso2 in NIID lesion cells, we next leveraged the same laser‐microdissection‐based proteomic workflow to define broader molecular alterations associated with NIID pathology. Unbiased proteomic comparison of lesion‐enriched sweat gland cells from 20 NIID patients with corresponding sweat gland cells from 6 healthy controls identified 326 differentially expressed proteins (DEPs), including 234 downregulated and 92 upregulated proteins in patient cells (Figure 6A and Table S2).

**FIGURE 6:**
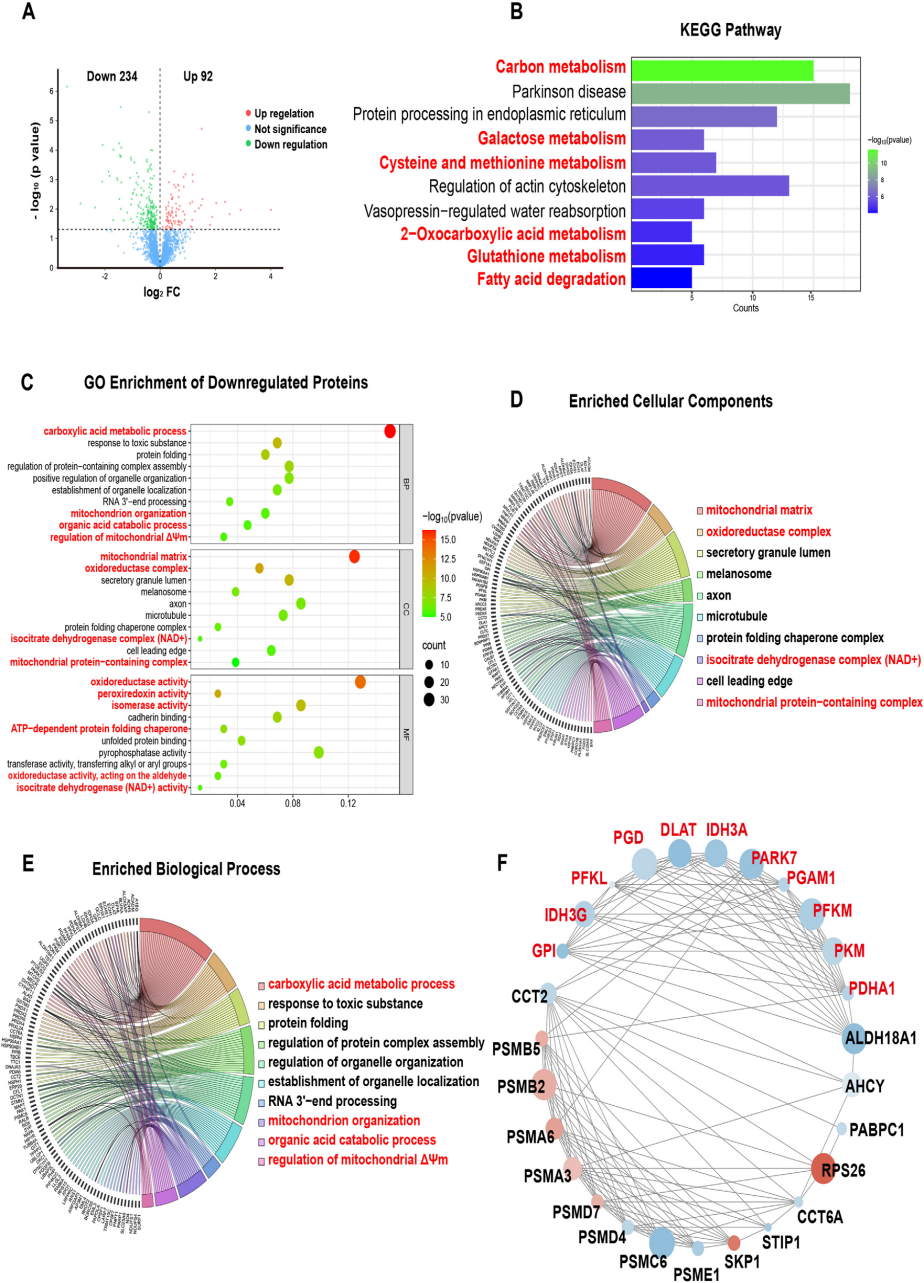
Proteomic analysis of sweat gland cells from NIID patients. (A) Volcano plot illustrating the differentially expressed proteins (DEPs) in sweat gland cells from NIID patients versus healthy controls. A total of 92 proteins were significantly upregulated (red dots) and 234 were significantly downregulated (green dots). The *x*‐axis represents the log_2_(fold change), and the *y*‐axis represents the ‐log_10_(*P*‐value). (B) Kyoto Encyclopedia of Genes and Genomes (KEGG) pathway enrichment analysis of all DEPs. Multiple pathways related to energy metabolism were significantly dysregulated (highlighted in red). Statistical significance was assessed using Fisher's exact test, with *P* < 0.05 considered significant. (C–E) Gene Ontology (GO) enrichment analysis of the downregulated proteins. The results significantly point to mitochondrial dysfunction and aberrant energy metabolism (highlighted in red), with all terms ranked by their statistical significance (P‐value). (D) A bubble plot of the GO enrichment analysis provides an overview of the most highly enriched terms across the Biological Process (BP), Cellular Component (CC), and Molecular Function (MF) categories. (E,F) are chord diagrams that respectively detail the most significant enriched terms within CC and BP, and list the specific proteins involved. (F) A significant protein‐protein interaction complex identified from the DEPs using the MCODE algorithm. Nodes are colored according to their expression status: red for upregulated proteins and blue for downregulated proteins. A subnetwork of proteins functionally related to mitochondrial energy metabolism is encircled with a red border. The size of each node is proportional to the statistical significance of the differential expression.

Functional enrichment analysis of all DEPs revealed significant dysregulation of pathways critical for cellular energetics, including Carbon metabolism and the TCA cycle (Figure 6B). Gene Ontology (GO) analysis of the more numerous downregulated proteins pointed strongly to mitochondrial dysfunction, with enriched terms including carboxylic acid metabolic process and localization to the mitochondrial matrix and oxidoreductase complexes (Figure 6C–E). A protein‐protein interaction (PPI) network analysis further identified a densely connected module of downregulated proteins, including the key metabolic enzymes PGD, DLAT, and IDH3A (Figure 6F).

To determine whether the protein interaction network engaged by PolyGN2C‐iso2 overlaps with or differs from the previously reported uN2CpolyG interactome, we performed FLAG immunoprecipitation followed by mass spectrometry in U2OS cells expressing empty vector, PolyG(14×)N2C‐iso2‐Flag, or pathogenic PolyG(64×)N2C‐iso2‐Flag. Compared with vector control and PolyG(14×)N2C‐iso2, pathogenic PolyG(64×)N2C‐iso2 enriched a set of candidate associated proteins, including factors involved in mitochondrial transport and metabolism, protein quality control, and DNA damage response (Figure S5A–C). Functional enrichment analysis showed that these proteins were mainly enriched in pathways related to ubiquitin‐mediated proteolysis, autophagy, mitophagy, and neurodegenerative diseases. In addition, cellular component analysis suggested an association with mitochondrial outer membrane‐related structures (Figure S5D–G). These findings support the notion that mitochondrial dysfunction and impaired protein homeostasis may represent important mechanisms in the development and progression of NIID.

We next compared the PolyGN2C‐iso2 FLAG‐IP‐MS dataset with previously reported uN2CpolyG interactomes. Several known uN2CpolyG‐interacting proteins were also detected in the PolyGN2C‐iso2‐associated proteome (Table S3). This suggests that part of the uN2CpolyG‐associated interaction network is conserved in PolyGN2C‐iso2. Meanwhile, additional candidate proteins, including SLC25A3, SLC25A10, WARS2, MAP2K3, SPTBN1, TDP1, and SEPTIN11, were enriched in the PolyG(64×)N2C‐iso2 pulldown (Table S3). These candidates may reflect PolyGN2C‐iso2‐biased molecular associations, possibly related to the longer NOTCH2NLC‐derived sequence and conserved NOTCH2NL‐related motifs present in PolyGN2C‐iso2. Together, these PolyGN2C‐iso2‐centered proteomic data indicate that PolyGN2C‐iso2 and uN2CpolyG may share partially overlapping interaction networks, while also retaining distinct features.

### 2.7 PolyGN2C‐iso2 Expression Drives Mitochondrial Abnormalities

These proteomic data implicated mitochondrial dysfunction as a core pathogenic mechanism. We first validated this in patient tissues by analyzing muscle biopsies from NIID patients, which revealed classic features of mitochondrial myopathy, including ragged‐red fibers (RRFs), intense subsarcolemmal SDH activity, and COX‐negative fibers (Figure 7A).

**FIGURE 7:**
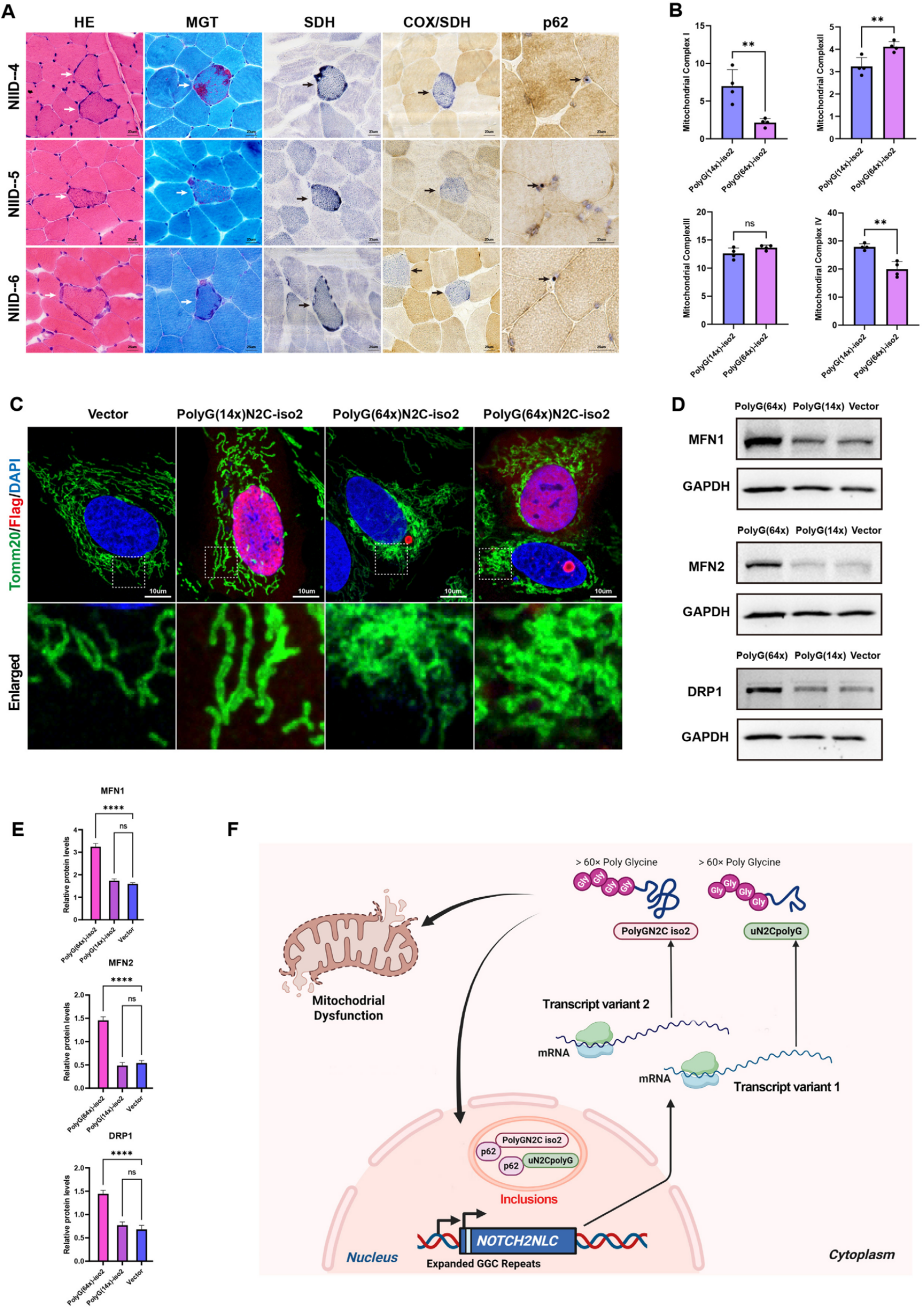
PolyG(64×)N2C‐iso2 protein induces mitochondrial abnormalities. (A) Histopathological analysis of muscle biopsies from NIID patients (*n* = 3). Staining includes: hematoxylin and eosin (H&E), showing abnormal muscle fibers with basophilic edges and mild granular changes (arrow); modified Gomori trichrome (MGT), showing ragged‐red fibers (arrow); succinate dehydrogenase (SDH), showing ragged‐blue fibers indicative of mitochondrial accumulation (arrow); cytochrome c oxidase (COX)/SDH dual staining, showing COX‐deficient fibers indicative of respiratory chain defects (arrow); and p62 immunohistochemistry, showing nuclear inclusions indicative of impaired protein clearance (arrow). Scale bars, 25 µm. (B) Quantification of mitochondrial respiratory chain complex levels in U2OS cells transfected with TV2‐PolyG‐Flag (14× or 64×) constructs. (C) Representative immunofluorescence of U2OS cells transfected as in (B). Cells were stained for mitochondria (Tomm20, green), the expressed protein (FLAG, red), and nuclei (DAPI, blue). Note the fragmented mitochondrial network in the PolyG(64×)N2C‐iso2‐expressing cell. Scale bar, 10 µm. (D and E) Immunoblots (D) and corresponding quantification (E) of mitochondrial dynamics proteins MFN1, MFN2, and DRP1 in U2OS cells transfected as in (B). (F) Proposed model for the molecular pathology of NIID. GGC repeat expansions in *NOTCH2NLC* produce two toxic proteins: uN2CpolyG (from TV1) and PolyGN2C‐iso2 (from TV2). These proteins co‐aggregate into intranuclear inclusions and drive mitochondrial dysfunction, leading to cellular pathology. Data are presented as mean ± SEM; *n* = 4 (B) or *n* = 3 (D,E) independent experiments per group. Statistical significance was determined by two‐tailed Student's t‐test (for comparisons between two groups in B) or one‐way ANOVA with Tukey's multiple comparisons test (for comparisons among three groups in E). ^**^ *P* < 0.01, ^****^ *P* < 0.0001.

To determine if PolyGN2C‐iso2 directly causes this pathology, we expressed PolyG(64×)N2C‐iso2 in U2OS cells. Pathogenic PolyGN2C‐iso2 expression led to a significant reduction in the levels of mitochondrial respiratory chain complexes I and IV (Figure 7B), indicating impaired electron transport chain function. This bioenergetic defect was accompanied by severe mitochondrial fragmentation, contrasting with the normal tubular network observed in control cells (Figure 7C). Mechanistically, this fragmentation was associated with a profound dysregulation of mitochondrial dynamics, evidenced by a marked upregulation of the fission protein DRP1 and the fusion proteins MFN1 and MFN2 (Figure 7D,E).

These data indicate that PolyGN2C‐iso2 can induce mitochondrial dysfunction, cause both bioenergetic failure and morphological defects, and provide a mechanistic link between this pathogenic protein and the mitochondrial pathology observed in NIID patients.

## 3 Discussion

Our study refines the molecular understanding of neuronal intranuclear inclusion disease by supporting a dual‐proteinopathy model involving two distinct NOTCH2NLC‐derived proteins, namely uN2CpolyG and PolyGN2C‐iso2. This revised model is supported by two key lines of evidence. First, using isoform‐discriminating monoclonal antibodies and antibody‐independent targeted proteomics, we provide patient‐tissue evidence that PolyGN2C‐iso2 is a component of the characteristic p62‐positive intranuclear inclusions. Second, and more critically, we establish the autonomous pathogenic capacity of PolyGN2C‐iso2 through a novel mouse model, showing that its sole expression is sufficient to recapitulate the core pathological, radiological, cognitive, and neuropsychiatric hallmarks of NIID. This expanded pathogenic framework suggests that future therapeutic strategies may need to consider both uN2CpolyG and PolyGN2C‐iso2 to achieve maximal efficacy.

Characterization of PolyGN2C‐iso2 offers critical insight into the neuropathological complexity of NIID. The prevailing pathogenic model of NIID has centered on the uN2CpolyG protein, translated from *NOTCH2NLC* transcript variant 1 [7, 20]. However, several histopathological observations have hinted at a more complex protein composition of NIID inclusions. For instance, a disparity in immunohistochemical positivity between the antibody 4D12 (89.5%) and the uN2CpolyG‐specific antibody 4C4 (56.9%) in NIID brain tissue [21]. In addition, Wang et al. reported that a commercial antibody against NOTCH2NLC isoform 2 detected p62‐positive intranuclear immunoreactivity in NIID skin samples [23]. This observation is important; nevertheless, NOTCH2NLC belongs to the highly homologous human‐specific NOTCH2NL paralogue family, including NOTCH2NLA, NOTCH2NLB, NOTCH2NLC, and NOTCH2NLR [24], and commercial NOTCH2NL antibodies directed against shared family epitopes may not fully distinguish transcript variant 2‐derived PolyGN2C‐iso2 from closely related NOTCH2NL‐family proteins. To overcome this limitation, we developed two monoclonal antibodies, M11F6 and M38D5, targeting distinct regions of PolyGN2C‐iso2. Using these isoform‐discriminating antibodies, we detected PolyGN2C‐iso2 in p62‐positive intranuclear inclusions in NIID patient tissues and observed its extensive co‐localization with uN2CpolyG. Importantly, this antibody‐based evidence was further supported by targeted proteomic analysis of laser‐microdissected p62‐positive sweat gland lesion cells, in which a PolyGN2C‐iso2‐discriminating surrogate peptide was detected. This antibody‐independent evidence strengthens the conclusion that PolyGN2C‐iso2 is a component of NIID pathological inclusions. This dual‐protein composition of inclusions is recapitulated in our cellular models, where pathogenic PolyG(64×)N2C‐iso2 forms aggregates that co‐localize with uN2CpolyG. The consistent co‐localization of both proteins in patient tissues and cellular systems supports a dual‐protein pathogenic model, in which uN2CpolyG and PolyGN2C‐iso2 jointly contribute to NIID pathogenesis. These findings refine the current understanding of inclusion body composition and establish the contribution of PolyGN2C‐iso2 to NIID pathology.

Beyond its role as a co‐component of inclusions, our work supports PolyGN2C‐iso2 as an autonomous pathogenic driver through a novel mouse model that captures several NIID‐like pathological, radiological, and behavioral features [12, 20]. This AAV‐based model, expressing only PolyG(108×)N2C‐iso2, demonstrates that this protein alone is sufficient to drive core aspects of NIID pathology. The utility of this model is demonstrated across multiple dimensions. Notably, this model exhibits white matter abnormalities and ventricular enlargement, hallmarks of the human disease that have been difficult to fully replicate in prior models [26, 27]. In addition to these morphological changes, the mice displayed spontaneous seizure‐like phenotypes, a clinical feature of NIID that remains challenging to reproduce in experimental settings [28]. Furthermore, the model exhibits a disease progression that more closely mirrors the chronic course of the human condition [29]. Systemically, it captures an important multisystem feature of NIID, with widespread intranuclear inclusions observed throughout the central nervous system and peripheral organs [10]. By integrating these pathological, systemic, and clinical dimensions, this model supports the autonomous pathogenic potential of PolyGN2C‐iso2 and provides a translationally relevant system for future mechanistic and therapeutic exploration. We do not infer from this model that PolyGN2C‐iso2 is quantitatively equivalent to, or more deleterious than, uN2CpolyG. A direct uN2CpolyG‐versus‐PolyGN2C‐iso2 comparison will require parallel animal studies using matched AAV design, promoter, delivery route, mouse background, expression level, repeat length, and analysis time point. Future studies using matched AAV‐based iso1‐ and iso2‐expression models will be necessary to determine their relative pathogenic potency and contributions to disease burden.

Mechanistically, our findings support a convergent but non‐identical pathogenic model for PolyGN2C‐iso2 and uN2CpolyG. Although these two proteins are structurally distinct and may engage partially different molecular interactors, they may converge on overlapping downstream disturbances, among which mitochondrial dysfunction appears to be an important pathogenic pathway. Our data suggest that PolyGN2C‐iso2 may perturb cellular homeostasis through mechanisms that partly overlap with those previously associated with uN2CpolyG [14, 30]. One pathway implicated is autophagy, the cell's primary system for clearing toxic protein aggregates [31, 32]. We observed that PolyGN2C‐iso2 expression impairs autophagic flux, evidenced by the accumulation of LC3‐II and p62. This disruption may further promote the buildup of toxic polyG species [33]. In parallel, our proteomic and functional analyses point to mitochondrial dysfunction as an important downstream alteration, with PolyGN2C‐iso2 expression associated with respiratory chain defects and mitochondrial network fragmentation. Although the precise upstream mechanism remains to be defined, our proteomic and autophagy‐related findings suggest that PolyGN2C‐iso2 may contribute to abnormalities in mitochondrial dynamics through disrupted proteostasis, impaired mitochondrial quality control, and mitochondrial stress. The PolyGN2C‐iso2 FLAG‐IP‐MS analysis further supports this view. Our interactomic profiling revealed that PolyGN2C‐iso2 partially shares its interaction network with uN2CpolyG (e.g., Ku70/80) [12], but additionally recruits unique candidate proteins related to the outer mitochondrial membrane, mitophagy, and ubiquitin‐mediated proteolysis. This interactomic divergence suggests that while both pathogenic polyG proteins converge on proteostasis disruption [15], PolyGN2C‐iso2 may exert an isoform‐biased insult that specifically drives the downstream mitochondrial fragmentation and bioenergetic failure. This mitochondrial disturbance may help explain the white matter pathology observed in our model, as proper myelination depends on mitochondrial integrity [34, 35]. This finding is also in line with previous clinical reports describing a striking overlap in neuroimaging and clinical features between a subset of NIID patients and those with MELAS, a canonical mitochondrial disease, supporting the relevance of mitochondrial dysfunction in NIID pathogenesis [36]. Furthermore, the perinuclear accumulation of PolyGN2C‐iso2 aggregates causes pronounced nuclear compression and deformation, an observation that aligns with reported lamina disruption by other polyG proteins [13, 37]. This finding may represent an additional, convergent pathogenic mechanism. Collectively, our results indicate that while structurally distinct, PolyGN2C‐iso2 and uN2CpolyG converge on several essential cellular homeostatic pathways. Together, these alterations may contribute to neurodegeneration through a multi‐hit model of cellular injury involving autophagy, mitochondrial function, and nuclear integrity.

While toxic gain‐of‐function mechanisms are established in NIID pathogenesis, our findings prompt consideration of a more complex disease model that may also incorporate loss‐of‐function components [12, 15]. The *NOTCH2NLC* gene, which encodes PolyGN2C‐iso2, plays important roles in human cortical development [24, 25]. Previous work has demonstrated that GGC repeat expansion in transcript variant 1 suppresses translation of its primary open reading frame, suggesting haploinsufficiency as one pathogenic component [1, 13]. Our study extends this concept to transcript variant 2, where AlphaFold3 structural predictions indicate that the expanded polyG tract likely disrupts the native conformation of PolyGN2C‐iso2, potentially compromising its physiological functions. These parallel findings across both transcript variants suggest that GGC repeat expansions may subvert *NOTCH2NLC* function through a dual mechanism: introducing toxic polyG‐containing proteins while simultaneously disrupting the normal functions of the native *NOTCH2NLC* isoforms.

However, several limitations of our study should be acknowledged. While our AAV model supports the independent pathogenic capacity of PolyGN2C‐iso2, it does not recapitulate the dual‐protein environment where both PolyGN2C‐iso2 and uN2CpolyG coexist in patients. Consequently, the potential interactions between these proteins and their relative contributions to disease burden remain to be determined. Additionally, our proposition of a loss‐of‐function mechanism, while supported by structural predictions, remains hypothetical and requires experimental validation. Future studies should aim to define the specific physiological consequences of *NOTCH2NLC* haploinsufficiency and develop models co‐expressing both toxic proteins to dissect their complex interplay. A remaining limitation of this study is that PolyGN2C‐iso2‐positive inclusions have not yet been directly confirmed in human NIID brain tissue. Although skin biopsy is widely used for the diagnosis of NIID, and our skeletal muscle findings suggest that the coexistence of PolyGN2C‐iso2 and uN2CpolyG is not restricted to the skin, direct evidence from the central nervous system is still lacking. Supporting our findings, targeted proteomic analysis of laser‐microdissected p62‐positive sweat gland lesion cells detected PolyGN2C‐iso2 at the protein level in an antibody‐independent manner. However, the estimated proportions of PolyGN2C‐iso2 and uN2CpolyG were derived from Skyline‐extracted signals of distinct surrogate peptides without calibration using isotope‐labeled internal standards. These estimates should therefore be interpreted as relative peptide‐signal measurements rather than absolute protein abundance or copy‐number ratios. Future studies using well‐preserved postmortem brain tissues from genetically confirmed NIID patients, combined with isotope‐labeled targeted proteomics, will be important for clarifying the CNS distribution of PolyGN2C‐iso2‐positive inclusions and their contribution to NIID pathology. Addressing these questions will be essential for developing a comprehensive understanding of NIID and designing therapeutic strategies that target the full spectrum of its molecular drivers.

## 4 Conclusion

In conclusion, our findings support an expanded disease model in which NIID involves both uN2CpolyG and PolyGN2C‐iso2 as NOTCH2NLC‐derived pathogenic proteins (Figure 7F). This updated pathogenic framework carries important therapeutic implications: targeting only one of these toxic proteins may be insufficient to halt disease progression. We therefore propose that future therapeutic efforts should aim to simultaneously eliminate both pathogenic proteins to achieve maximal clinical benefit. The development of strategies enabling such dual targeting represents a pivotal next step for the field.

## 5 Methods

### 5.1 Study Design

The objective of this study was to investigate the pathogenic role of PolyGN2C‐iso2, a protein encoded by *NOTCH2NLC* transcript variant 2. The experimental design comprised three main components. First, to determine the presence of PolyGN2C‐iso2 in patient pathology, we developed novel monoclonal antibodies and performed immunohistochemical analyses on biopsies from NIID patients. Second, to establish the autonomous pathogenic capacity of PolyGN2C‐iso2 in vivo, we generated a novel AAV‐based mouse model exclusively expressing the human pathogenic protein and conducted neuropathological, neuroimaging, and behavioral assessments [29]. Third, to elucidate underlying molecular mechanisms, we performed unbiased proteomic profiling on patient‐derived cells, followed by functional validation of key pathways in cellular models.

For all experiments, sample sizes were determined based on previous studies and are detailed in the corresponding figure legends. Investigators were blinded to group assignments during data acquisition and analysis. All procedures involving human subjects were approved by the local Institutional Review Board, and all animal experiments were conducted in accordance with approved protocols from the Institutional Animal Care and Use Committee. Further detailed methods are provided in the Supplementary Information.

### 5.2 Statistics

All statistical analyses were performed using GraphPad Prism 9 (GraphPad Software). Data are presented as mean ± standard error of the mean (SEM). Normality and homogeneity of variances were assessed using the Shapiro‐Wilk test and the Brown‐Forsythe test, respectively. For comparisons between two groups, the two‐tailed unpaired Student's t‐test was used. For comparisons among multiple groups, one‐way or two‐way analysis of variance (ANOVA) was employed, followed by Tukey's multiple comparisons test. Non‐parametric tests were used if data did not meet the assumptions for parametric tests. Survival data were analyzed using Kaplan‐Meier curves. A *P*‐value < 0.05 was considered statistically significant. Significance levels are denoted as: ^*^ *P* < 0.05, ^**^ *P* < 0.01, ^***^ *P* < 0.001, and ^****^ *P* < 0.0001. Specific statistical tests, sample sizes (n), and experimental replicates are detailed in the corresponding figure legends. Investigators were blinded to group allocation during data analysis.

## Author Contributions

Conceptualization: XW, ZZ, KZ, WM. Methodology: XW, ZZ, MJK, KZ, WM. Investigation: KZ, WM, ZW, HN, YZ, TZ, ZD, LL, YL, SY, WS, XD, PG, YP. Data Curation: KZ, HT, YZ, AW, SS, HP. Formal Analysis: KZ, WM, ZW, HN, YZ, TZ, ZD, LL, YL, SY, WS, XD, PG, YP. Writing – original draft: KZ. Writing – review & editing: XW, ZZ, MJK, KS. Supervision: XW, ZZ, MJK. Funding acquisition: KZ, ZZ, HT, MJK.

## Funding

National Natural Science Foundation of China grant 82201562 (K.Z.), National Natural Science Foundation of China grant 82271903 (Z.Z.), Beijing Yicheng Cooperation Development Public Welfare Fund grant YCXJ‐JZ‐2023‐017 (K.Z.), Beijing Yicheng Cooperation Development Public Welfare Fund grant YJXJ‐JZ‐2021‐0014 (H.T.), Chinese Institute for Brain Research, Beijing core grant and Chinese Academy of Medical Sciences Innovation Fund for Medical Sciences grant 2019‐I2M‐5‐015 (M.K.), Beijing Talent Program, co‐funded by Beijing Tiantan Hospital and the Chinese Institute for Brain Research, Beijing (K.Z.)

## Ethics Statement

Human biological samples (skin and muscle) were obtained during routine clinical procedures. Written informed consent was obtained from all patients or their legal representatives upon hospital admission, permitting the use of their biological specimens for scientific research. The collection and use of these samples were conducted in accordance with the Declaration of Helsinki and the institutional guidelines of Beijing Tiantan Hospital, Capital Medical University. All animal experiments were approved by the Institutional Animal Care and Use Committee of Beijing GeneCradle Technology Co., Ltd (approval No. JL‐IACUC‐20240317‐09E) and conducted in accordance with institutional guidelines.

## Consent

Written informed consent was obtained from all patients and healthy controls, or their legal representatives, for the use of clinical information and biological samples in this study.

## Conflicts of Interest

The authors declare no conflicts of interest.

## Supporting information




**Supporting File 1**: advs76702‐sup‐0001‐SuppMat.docx.

**Supporting File 2**: advs76702‐sup‐0002‐MovieS1.mp4.

## Data Availability

The data that support the findings of this study are available in the supplementary material of this article.
