# Supplementary material for: A Second Pathogenic Protein, PolyGN2C‐iso2, Reveals a Dual‐Protein Pathology in Neuronal Intranuclear Inclusion Disease
Source: Adv Sci (Weinh). 2026 Jul 23:e76702. Online ahead of print. doi: 10.1002/advs.76702 (PMC13393261; doi:10.1002/advs.76702)
Supplement: Supplementary file 1 — Supporting File 1: advs76702‐sup‐0001‐SuppMat.docx. [file ADVS-9999-e76702-s001.docx]

Supporting Information

**A second pathogenic protein, PolyGN2C-iso2, reveals a dual-protein pathology in neuronal intranuclear inclusion disease**

**Running title: PolyGN2C-iso2 in NIID**

*Kang Zhang^1†^, Wenhao Ma^2, 3, 7†^, Yi Zhou^1^, Zhijie Wu^2^, Pan Gao^2^, Hongze Niu^2^, Hongfei Tai^1^, Tianyi Zhao^2^, Zheyue Dong^2^, Li Li^2^, Yan Zhang^2^, An Wang^1^, Si Shen^1^, Yueyang Li^4, 5, 6^, Sifei Yu^4, 5, 6^, Yan Peng^4, 5, 6^, Wang Sheng^7^, Xiaoyan Dong^2^, Hua Pan^1^, Kaibin Shi^1, 8^, Magdalena J. Koziol^4, 5, 6^*, Xiaobing Wu^2^*,* *Zaiqiang Zhang^1^**

*Corresponding author:

Zaiqiang Zhang, Email: [ttyy0142011@126.com](mailto:ttyy0142011@126.com)

Xiaobing Wu, Email: [wuxiaobing@bj-genecradle.com](mailto:wuxiaobing@bj-genecradle.com)

Magdalena J. Koziol, Email: [mjk@cibr.ac.cn](mailto:mjk@cibr.ac.cn)

**Supplementary Materials and Methods**

**Human samples**

Human skin, muscle, and peripheral venous blood samples were collected from 26 patients with NIID and 7 healthy controls under a protocol approved by the Ethics Committee of Beijing Tiantan Hospital, Capital Medical University. Written informed consent was obtained from all participants or their legal guardians. The samples from the 7 healthy controls were obtained from individuals during surgical debridement for limb trauma. All control individuals had no known history of neurological or muscular diseases.

**Cell cultures**

HEK293T cells (China National Collection of Authenticated Cell Cultures, GNHu44) were cultured in high-glucose DMEM medium (Gibco, C11995500BT), while U2OS cells (China National Collection of Authenticated Cell Cultures, SCSP-5030) were cultured in McCoy’s 5A medium (Gibco, 16600-082). Both media were supplemented with 10% fetal bovine serum (FBS) and 100 U/ml penicillin - 100 μg/ml streptomycin. All cell lines were routinely tested negative for mycoplasma contamination. All cells were maintained in a humidified incubator at 37°C with 5% CO₂.

**Plasmid Constructs for Cell Culture**

The TV2-PolyG-Flag expression plasmid was constructed based on the pLVX-Tight-Puro backbone (Takara, 632162). It contains the coding sequence (CDS) of *NOTCH2NLC* transcript variant 2 with a C-terminal FLAG tag driven by a CMV promoter. To generate polyglycine expansions, (GGC)₁₄ or (GGC)₆₄ repeats were inserted into the CDS. For dual-expression experiments, the TV1-PolyG-HA-TV2-PolyG-Flag plasmid was generated using the pAAV-GFP backbone (Addgene, 32395). This construct utilizes a CAG promoter to drive the expression of the *NOTCH2NLC* variant 1 5' UTR (tagged with HA) linked via a P2A peptide to the variant 2 CDS (tagged with FLAG). All constructs were verified by Sanger sequencing.

**AAV Vector Construction and Production**

For *in vivo* studies, the CDS of human *NOTCH2NLC* transcript variant 2 containing either 14 [(GGC)₁₄] or 108 [(GGN)₁₀₈] repeats were commercially synthesized and cloned into the pUC57 vector. These fragments were subsequently subcloned via *Kpn*I/*Eco*RI digestion into an AAV transfer plasmid backbone containing wild-type AAV2 inverted terminal repeats (ITRs), a CMV promoter, and a bovine growth hormone (BGH) polyadenylation signal. This process yielded the final transfer plasmids: pITR-CMV-NOTCH2NLC_iso2-BGH (control) and pITR-CMV-polyG_NOTCH2NLC_iso2-BGH (disease model).

Recombinant AAV9 (rAAV9) vectors were produced by transient triple-transfection of HEK293T cells. Cells were co-transfected with the transfer plasmid, an AAV2 Rep/AAV9 Cap plasmid, and an adenovirus helper plasmid (pHelper). Cells were harvested and lysed 72 hours post-transfection. Viral particles were purified from the lysate using CsCl gradient ultracentrifugation (100,000 × *g*, 18 h, 4°C). The purity of the viral preparations was confirmed by silver-stained SDS-PAGE. Genomic titers were quantified via TaqMan qPCR targeting the CMV promoter region using the following primers and probe: Forward, 5’-CGTTTAGTGAACCGTCAGATCG-3’; Reverse, 5’-GGTCCCGGTGTCTTCTATGG-3’; Probe, 5’-(TAMRA)-CCTGGAGACGCCATCCACGCTGT-(FAM)-3’.

**Antibody production**

To generate monoclonal antibodies against M38D5 (WRSGCAARPPRM) and M11F6 (RDGYEPCVNEG), female BALB/c mice (6–8 weeks old) were immunized subcutaneously with KLH-conjugated antigens emulsified in complete Freund’s adjuvant (primary, 60 μg), followed by four boosters (30 μg each) in incomplete Freund’s adjuvant every two weeks. Three days before fusion, mice with positive serum responses received an intraperitoneal boost (50 μg antigen). Splenocytes were fused with Sp2/0 myeloma cells; resulting hybridomas cultured and screened via ELISA. Positive clones underwent two rounds of soft agar cloning. Monoclonal antibodies were produced by injecting hybridoma cells intraperitoneally into pristane-pretreated mice. Ascites was purified using Protein A/G affinity chromatography, and the purified antibodies were stored at -80°C. The final stock concentrations were 2.2 mg/mL for M38D5 and 2.7 mg/mL for M11F6. Animal experiments complied with Chinese ethical guidelines. Antibodies were prepared by BGI Genomics Co., Ltd **(Table S4).**

**Molecular modeling and dynamics simulation of uN2CpolyG and PolyGN2C-iso2**

The three-dimensional structures of uN2CpolyG and PolyGN2C-iso2 proteins predicted by AlphaFold3 (based on the model released on May 8, 2024, via the AlphaFold Server platform) were subjected to molecular dynamics simulations using GROMACS v2019.5. Systems (Groningen Machine for Chemical Simulations) were parameterized with the CHARMM27 force field ^1,2^ and solvated in a TIP3P water model within an orthorhombic box maintaining 1.2 nm minimum distance from protein surfaces, with Na^+^/Cl^−^ ions added for charge neutralization.

Energy minimization was conducted via steepest descent algorithm (5000 steps) to resolve steric clashes. Subsequent equilibration involved sequential NVT (constant number of particles, volume, and temperature) and NPT (constant number of particles, pressure, and temperature) phases: initial 100 ps NVT equilibration at 300 K employed the V-rescale thermostat with backbone restraints, followed by 100 ps NPT equilibration at 1 bar using Parrinello-Rahman pressure coupling.

Production simulations were performed for 50 ns with 2 fs timestep under periodic boundary conditions. Bond lengths were constrained by LINCS algorithm ^3,4^, while long-range electrostatic interactions were calculated using PME method with 1.0 nm cutoff for both Coulombic and van der Waals interactions ^5^. Trajectory data collected at 1 ps intervals were analyzed through Visual Molecular Dynamics (VMD) ^6,7^ for root-mean-square deviation (RMSD), residue fluctuation (RMSF), hydrogen bond dynamics, and secondary structure evolution. Final conformational states were visualized through PyMOL molecular graphics system.

**Repeat‑Primed Polymerase Chain Reaction (RP‑PCR)**

RP-PCR was performed to detect the GGC repeat expansion in the NOTCH2NLC gene of patients with NIID. The PCR reaction system and cycling conditions were established based on previous studies ^8^. The primer sequences used for amplification were as follows:

NOTCH2NLC-RP-F: 5’-FAMGGCATTTGCGCCTGTGCTTCGGACCGT-3’,

M13-(GGC)₄(GGA)₂-R: 5’-CAGGAAACAGCTATGACCTCCTCCGCCGCCGCCGCC-3’,

M13-linker-R: 5’-CAGGAAACAGCTATGACC-3’.

**Development of the PolyGN2C-iso2 Mouse Model**

Pregnant C57BL/6 mice (Beijing Vital River) were housed under SPF conditions at Beijing GeneCradle Technology Co., Ltd. Neonates were randomly allocated to three groups (WT, PolyG(14×)N2C-iso2, PolyG(108×)N2C-iso2; *n*=7/group). The sample size was chosen based on previous NIID mouse model studies that demonstrated sufficient statistical power with 5-8 animals per group ^9^, and in accordance with the 3Rs principles for animal welfare. Mice were received bilateral intracerebroventricular injections of 7.5×10¹³ vg/kg rAAV9 vectors via stereotaxic delivery (33G Hamilton needle) within 24 hours postnatally. Longitudinal assessments included behavioral phenotyping, cerebral MRI, and multiorgan sampling at 14 weeks post-injection. All procedures complied with ARRIVE 2.0 guidelines under Institutional Animal Care and Use Committee (IACUC) approval (JL-IACUC-20240317-09E) at Beijing GeneCradle.

**Mouse Brain MRI Analysis**

At week 14, mice underwent MRI. Mice were anesthetized via intraperitoneal injection of 1.25% tribromoethanol solution (Nanjing Aibei Biotechnology, M2920). Upon confirmation of adequate anesthesia, mice were placed in a 7.0T MRI system (Bioclinscan，Bruker，Germany) for brain imaging. During image acquisition, a dedicated four-channel surface coil for the mouse head was used to obtain coronal images. The imaging parameters were set as follows: T2-weighted imaging (T2WI), repetition time/echo time (TR/TE) = 3180/41 ms, slice thickness = 0.5 mm, field of view (FOV) = 20 × 20 mm, and matrix resolution = 256 × 256.

**Mouse behavioral analysis**

**Morris water maze**

Spatial learning and memory were assessed using a Morris water maze consisting of a 1m-diameter pool filled with water maintained at 21±1°C and rendered opaque with non-toxic white paint. A submerged platform (15 cm diameter) was placed in the target quadrant. Mice underwent quadrant-based training with four trials per day for five consecutive days, with entry points randomized across trials. Each trial had a 60-second cutoff, and mice that found the platform were allowed to remain on it for 10 seconds before being returned to their home cages. On the sixth day, a probe test was conducted by removing the platform and allowing mice to swim freely for 60 seconds. During the probe test, we recorded the platform-crossing frequency and the percentage of time spent in the target quadrant.

**Open field test**

Mice were placed in a 40×40 cm arena and allowed to explore freely for 10 minutes under standard room lighting conditions. Their movement was tracked and analyzed using automated tracking software that divided the arena into 16 quadrants. Locomotor activity was analyzed with velocity thresholds (0.1 m/s differentiating ambulatory vs. exploratory movement). The apparatus was thoroughly cleaned with 75% ethanol and air-dried between sessions to eliminate olfactory cues.

**Elevated plus maze**

Anxiety-like behavior was assessed using an elevated plus maze under controlled illumination of 15-50 lux. Following Knight's methodology ^10^, we recorded arm entries over a 5-minute session. The percentage of open arm entries was calculated as (number of open arm entries / total arm entries) × 100%. General locomotor activity was assessed by counting the total number of arm entries.

**Electron Microscopy of Mouse Brains**

Brains from mice were fixed in 2% formaldehyde (Merck; F8775) and 2.5% glutaraldehyde (Merck; 354400), post-fixed in 1% osmiumtetroxide (Merck; 20816-12-0), dehydrated in gradient ethanol, embedded in epoxy resin and polymerized at 60°C for 24 hours. Ultrathin sections (80 nm thick) were obtained from the resin blocks and picked up on copper grids, stained with uranyl acetate and lead citrate. Finally, the ultrathin sections were observed trough CCD camera using transmission electron microscope (FEI Tecnai12, USA) at 80kv.

**Proteomic and Bioinformatic Analysis**

Skin tissue sections from patients with NIID and healthy controls were subjected to immunohistochemical staining for p62 to identify sweat gland cells containing characteristic intranuclear inclusions. These target cells were then precisely isolated using a laser microdissection system. The captured cellular proteins were subsequently processed and analyzed by liquid chromatography-tandem mass spectrometry (LC-MS/MS) for protein identification and quantification. Protein identification and quantification were performed using Spectronaut (Biognosys, version 18.2) with searches conducted against the UniProt Human database. Both peptide and protein identifications were filtered at a 1% false discovery rate (FDR). Differentially expressed proteins (DEPs) between NIID patients and healthy controls were identified based on a statistical significance threshold of P < 0.05. To elucidate the biological functions of the identified DEPs, Gene Ontology (GO) and Kyoto Encyclopedia of Genes and Genomes (KEGG) pathway enrichment analyses were conducted using the Metascape online platform (http://metascape.org). A protein-protein interaction (PPI) network was constructed by submitting the DEPs to the STRING database (https://string-db.org). The resulting network was then visualized and analyzed in Cytoscape software. To identify densely connected modules within the network, the Molecular Complex Detection (MCODE) plugin for Cytoscape was employed with the following default parameters: Degree Cutoff = 2, Node Score Cutoff = 0.2, K-Core = 2, and Max. Depth = 100. The top-ranked module with the highest MCODE score was selected and a sub-network was generated for further investigation of key protein clusters.

**Targeted Mass Spectrometry Analysis and Skyline-Based Data Processing**
Raw mass spectrometry data from patient samples were imported into Skyline for targeted extraction of NOTCH2NLC-derived pathogenic protein peptides. The patient proteomic datasets were generated from laser-microdissected p62-positive sweat gland cells from NIID skin biopsy specimens. Target protein sequences corresponding to uN2CpolyG and PolyGN2C-iso2 were added to the Skyline target list. Two isoform-discriminating surrogate peptides were selected for semi-quantitative comparison of the two pathogenic protein species: CWRSGCAARPPRMHCSVEMAMNPV for uN2CpolyG and CWRSGCAARPPRMCR for PolyGN2C-iso2. The specificity of these peptides was evaluated by in silico comparison against NOTCH2NLC isoforms and closely related NOTCH2NL paralogues, including NOTCH2NLA, NOTCH2NLB, and NOTCH2NLR sequences.

Skyline parameters were configured for DIA-based analysis of patient samples. In the Peptide Settings, the maximum number of missed cleavages was set to 4, and the peptide length range was set to 6–100 amino acids. Carbamidomethylation of cysteine was set as a fixed modification, and methionine oxidation was included as a variable modification when applicable. In the Transition Settings, the acquisition method was set to DIA, with the isolation scheme set to “Results only” for full-scan filtering. For MS1 filtering, “Isotope peaks included” was set to “Count”, and the precursor mass accuracy was set to 20 ppm. Retention time filtering was set to “Include all matching scans”. Extracted chromatographic peaks were manually inspected to confirm correct peak boundaries, consistent retention time, appropriate isotope distribution, and absence of obvious interfering signals. After peak integration, the Skyline “Total Area” value for each target peptide was exported for downstream analysis. The relative contribution of each pathogenic protein species was estimated by dividing the total peak area of the corresponding surrogate peptide by the combined total peak area of both selected surrogate peptides. Because isotope-labeled internal peptide standards were not used, this analysis was interpreted as a semi-quantitative comparison of surrogate peptide signals rather than an absolute protein abundance measurement or protein copy-number ratio.

For cell sample analysis, U2OS cells overexpressing TV2-PolyG-Flag with either 14× or 64× GGC repeats were analyzed by mass spectrometry in DDA mode. The raw DDA data were imported into Skyline, and chromatograms of selected representative peptides were extracted to support peptide detectability in the overexpression system. For cell sample analysis, the maximum number of missed cleavages was set to 3, and the peptide length range was set to 6–100 amino acids. In the Transition Settings, precursor charges were set to 2, 3, and 4, ion charges were set to 1 and 2; and the ion type was set to precursor ions for MS1-based chromatogram extraction. In the Full-Scan panel, “Isotope peaks included” was set to “Count”, the precursor mass analyzer was set to Orbitrap, the acquisition method was set to DDA, the product mass analyzer was set to Orbitrap, and retention time filtering was set to “Include all matching scans”. After peak integration, the “Total Area” for each target peptide was exported for downstream analysis.

**Immunofluorescence and Immunohistochemistry**

For immunofluorescence, human skin and mouse brain paraffin sections (5 μm) were baked at 60°C for 1 hour, deparaffinized, rehydrated, and underwent antigen retrieval in citrate buffer (pH 6.0) using a pressure cooker for 1 hour. Sections were permeabilized with 0.5% Triton X-100 in PBS for 1 hour and blocked with 5% BSA at room temperature for another hour. Cultured cells on glass coverslips were fixed directly with 4% paraformaldehyde for 30 minutes, washed, permeabilized with 0.5% Triton X-100 for 15 minutes, and similarly blocked with 5% BSA. All samples were incubated overnight at 4°C with primary antibodies. For the detection of PolyGN2C-iso2, custom-made mouse monoclonal antibodies (clones M38D5 and M11F6) were generated by BGI Genomics Co., Ltd. (Shenzhen, China). Detailed information on all primary antibodies and their specific dilutions is provided in Table S4. After washing, Alexa Fluor® 488- or 594-conjugated secondary antibodies were applied for 1.5 hours at room temperature in the dark. Samples were mounted with VECTASHIELD® Antifade Mounting Medium with DAPI (Vector Laboratories, H-1200) and imaged using a Zeiss LSM 880 confocal microscope.

For immunohistochemistry, sections of human skin and mouse brain, muscle, liver, and kidney underwent the same initial processing and antigen retrieval. Endogenous peroxidase activity was quenched with 3% H₂O₂ for 15 minutes, followed by 5% BSA blocking for 1 hour. Sections were incubated overnight at 4°C with rabbit anti-p62/SQSTM1 (Proteintech, 18420-1-AP; 1:800) or the aforementioned custom PolyGN2C-iso2 antibodies (dilutions listed in Table S4), washed, and incubated with HRP-conjugated goat anti-rabbit or anti-mouse secondary antibodies for 1 hour. DAB substrate was applied for ≤3 minutes with microscopic monitoring, and the reaction was stopped with water. Sections were counterstained with hematoxylin (50 s), rinsed, briefly dipped in 70% ethanol, dehydrated, cleared in xylene, and mounted with neutral resin. Imaging was performed using an Olympus BX53 microscope.

**Muscle pathology analysis**

Muscle specimens were processed through standardized protocols at Beijing Tiantan Hospital Pathology Department, with cryosectioning (7 μm) and histological staining procedures replicating our established methodology19. Tissue sections underwent H.E. staining for architectural evaluation, complemented by mitochondrial functional assays through MGT, SDH, and COX/SDH staining. All histological assessments adhered to consensus protocols ^11^.

**Western blot analysis**

Cells were lysed in RIPA buffer (Beyotime, P0013C) containing protease inhibitors (Roche, 11836170001), followed by centrifugation (12,000×g, 20 min, 4°C) to collect supernatant. Protein concentrations were quantified using BCA assay (Beyotime, P0012S), with samples denatured in 5× loading buffer (Beyotime, P0015) at 95°C for 10 min. Proteins separated on 15% SDS-PAGE (Vazyme, E305-01) were transferred to PVDF membranes via wet transfer. Membranes blocked with 5% skim milk/TBST (1 h, RT) were incubated overnight at 4°C with primary antibodies listed in Table S4. After TBST washes, HRP-conjugated secondary antibodies (Abcam, ab205719/ab205718, 1:1000;) were applied (1 h, RT). Protein bands were visualized using ECL (Vazyme, E423-01) on an iBright 1500 system, with GAPDH serving as loading control. Semi-quantitative analysis was performed by measuring target/GAPDH band intensities using ImageJ.

**Mitochondrial complex analysis**

Cells were cleaved with RIPA lysate (Beyotime, P0013C) and centrifuged at 4℃ for 10min to obtain the supernatant. The supernatant was aliquoted into five equal parts for measuring the enzymatic activities of Mitochondrial Complexes I, II, III, and IV using colorimetric assay kits (Solarbio, China; BC0515, BC3253, BC3245, BC0945) according to the manufacturer's protocols. The protein concentration of the supernatant was quantified using the BCA assay (Beyotime; P0012S), and the complex activity values were normalized to the total protein content for each sample.

**Reference**

1. Varadi M, Anyango S, Deshpande M, et al. AlphaFold Protein Structure Database: massively expanding the structural coverage of protein-sequence space with high-accuracy models. *Nucleic Acids Res* 2022;**50**:D439-D444.

2. Buck M, Bouguet-Bonnet S, Pastor RW, MacKerell AD, Jr. Importance of the CMAP correction to the CHARMM22 protein force field: dynamics of hen lysozyme. *Biophys J* 2006;**90**:L36-38.

3. Lu J, Chen M, Qin Y. Drug-induced cell viability prediction from LINCS-L1000 through WRFEN-XGBoost algorithm. *BMC Bioinformatics* 2021;**22**:13.

4. Hess B. P-LINCS: A Parallel Linear Constraint Solver for Molecular Simulation. *J Chem Theory Comput* 2008;**4**:116-122.

5. Chen L, Cruz A, Roe DR, et al. Thermodynamic Decomposition of Solvation Free Energies with Particle Mesh Ewald and Long-Range Lennard-Jones Interactions in Grid Inhomogeneous Solvation Theory. *J Chem Theory Comput* 2021;**17**:2714-2724.

6. Vieira IHP, Botelho EB, de Souza Gomes TJ, Kist R, Caceres RA, Zanchi FB. Visual dynamics: a WEB application for molecular dynamics simulation using GROMACS. *BMC Bioinformatics* 2023;**24**:107.

7. Humphrey W, Dalke A, Schulten K. VMD: visual molecular dynamics. *J Mol Graph* 1996;**14**:33-38, 27-38.

8. Sone J, Mitsuhashi S, Fujita A, et al. Long-read sequencing identifies GGC repeat expansions in NOTCH2NLC associated with neuronal intranuclear inclusion disease. *Nat Genet* 2019;**51**:1215-1221.

9. Liu Q, Zhang K, Kang Y, et al. Expression of expanded GGC repeats within NOTCH2NLC causes behavioral deficits and neurodegeneration in a mouse model of neuronal intranuclear inclusion disease. *Sci Adv* 2022;**8**:eadd6391.

10. Knight P, Chellian R, Wilson R, Behnood-Rod A, Panunzio S, Bruijnzeel AW. Sex differences in the elevated plus-maze test and large open field test in adult Wistar rats. *Pharmacol Biochem Behav* 2021;**204**:173168.

11. Udd B, Stenzel W, Oldfors A, et al. 1st ENMC European meeting: The EURO-NMD pathology working group Recommended Standards for Muscle Pathology Amsterdam, The Netherlands, 7 December 2018. *Neuromuscul Disord* 2019;**29**:483-485.

**Supplementary Figures & Legends**


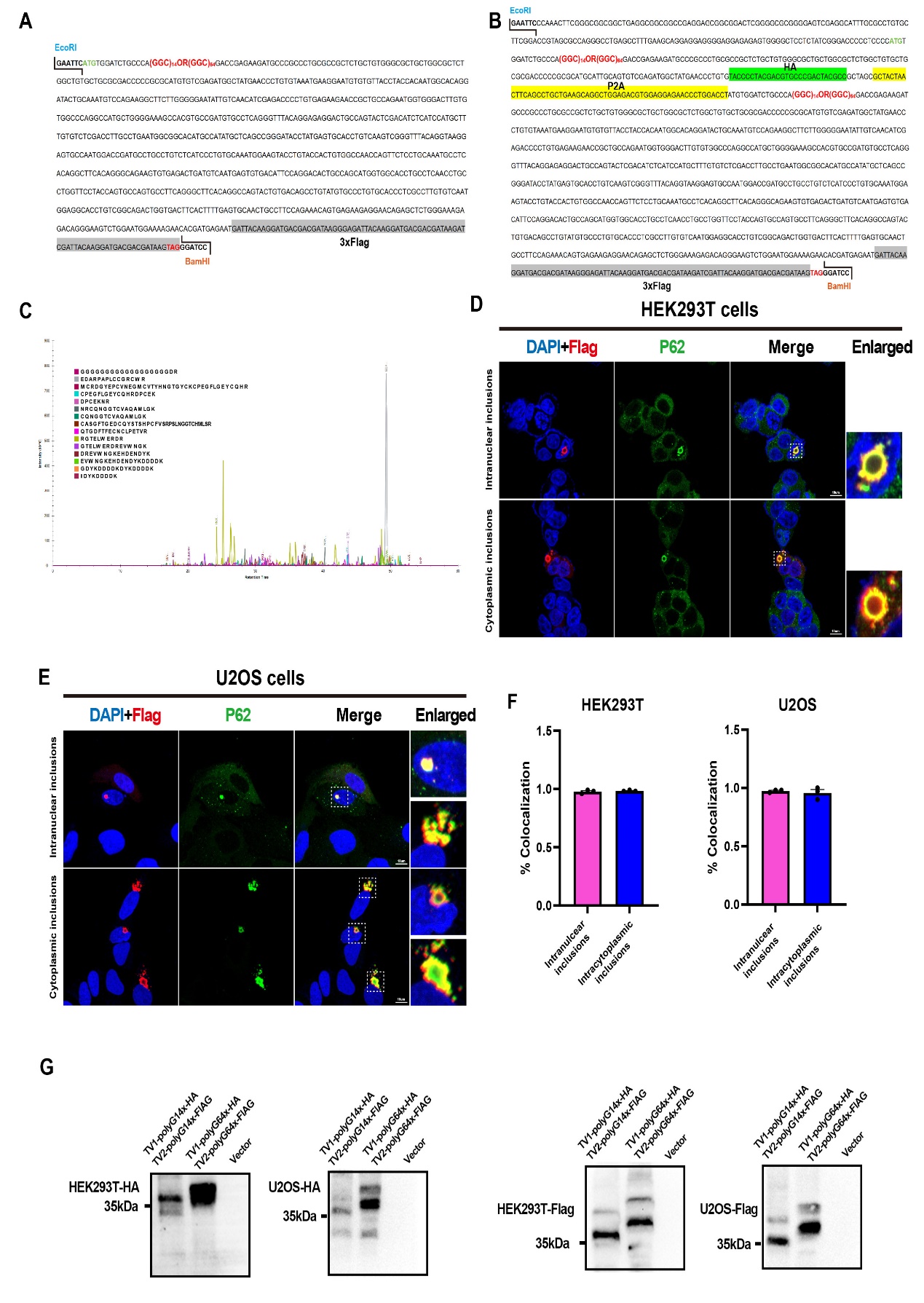


**Figure S1 Related to Figure 1**

**(A)** Schematic representation of key sequence elements within the TV2-PolyG-Flag construct. **(B)** Schematic representation of key sequence elements within the TV1-PolyG-HA-TV2-PolyG-Flag dual-expression construct. **(C)** Representative LC-MS/MS chromatogram from an excised gel band of U2OS cells expressing PolyG(64×)N2C-iso2-Flag. Identified peptides assigned to the TV2-derived PolyGN2C-iso2-3×Flag product are listed on the left. **(D-F)** HEK293T and U2OS cells were transfected with the TV2-PolyG-Flag construct containing 64×GGC repeats. Immunofluorescence performed 48h post-transfection showed that Flag-labeled PolyG(64×)N2C-iso2 inclusions co-localized with p62 in both the cytoplasm and nucleus. Quantification of co-localization between PolyG(64×)N2C-iso2 inclusions and p62 in the cytoplasm and nucleus. Data represent three independent biological replicates per cell line, with 100 inclusion-positive cells analyzed per replicate. **(G)** HEK293T and U2OS cells were transfected with the TV1-PolyG-HA-TV2-PolyG-Flag construct containing either 14×or 64×GGC repeats. At 48 h post-transfection, cell lysates were subjected to immunoblotting using anti-Flag and anti-HA antibodies to detect the expression of PolyGN2C-iso2 and uN2CpolyG, respectively.


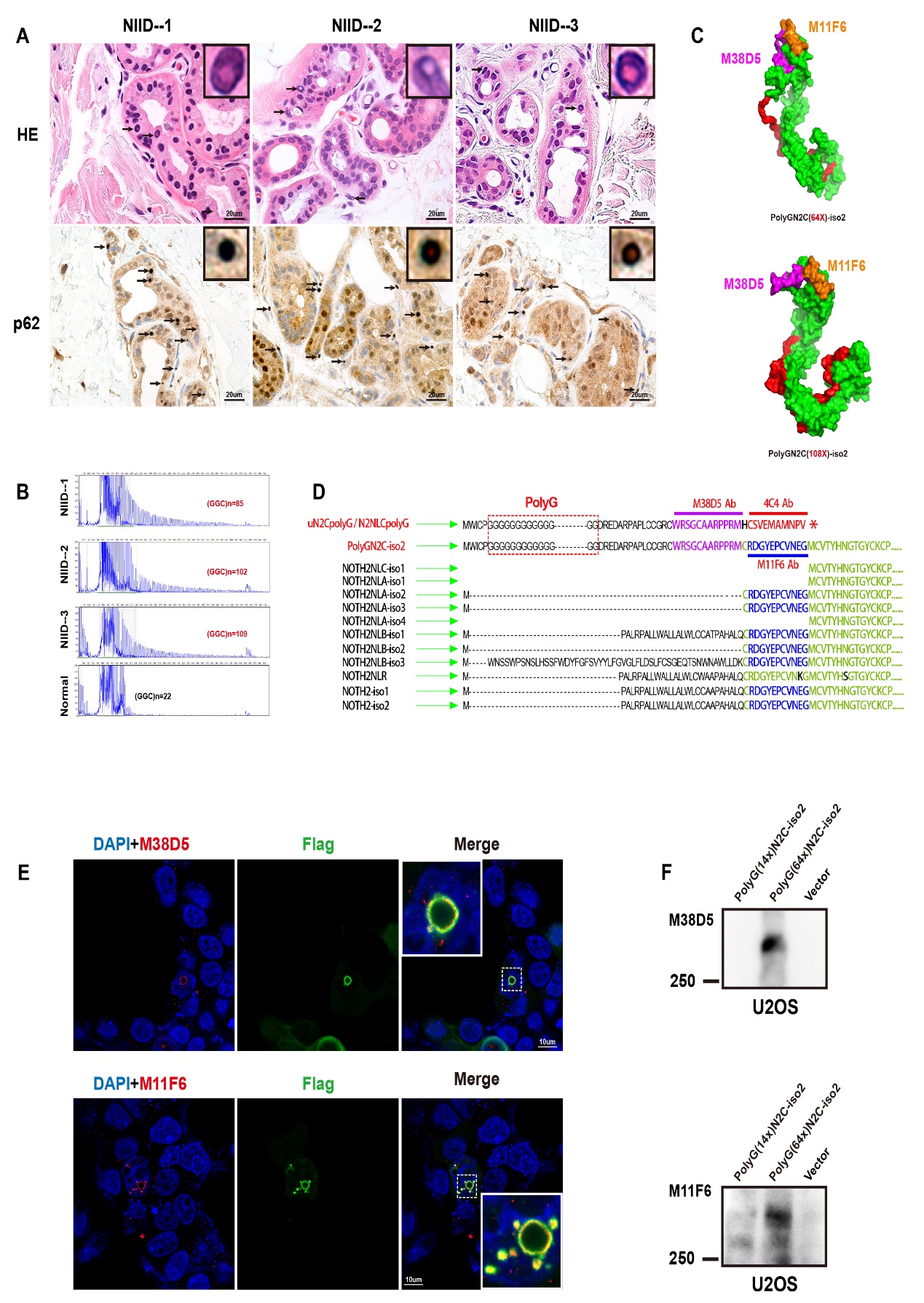


**Figure S2 Related to Figure 2**

**(A)** Representative HE-stained skin biopsy images (top) from three NIID patients included in Figure 2. Black arrows indicate eosinophilic intranuclear inclusions. Corresponding p62 immunohistochemistry images (bottom) show p62-positive intranuclear inclusions marked by black arrows. (**B**) Repeat-primed PCR (RP-PCR) analysis of the NOTCH2NLC gene showing expanded GGC repeats in all three NIID patients compared to the healthy control. (**C**) Predicted spatial locations of the M38D5 and M11F6 antibody epitopes on the three-dimensional structure of PolyG(64×)N2C-iso2 and PolyG(108×)N2C-iso2 proteins. (**D**) Amino acid sequence alignment of proteins encoded by NOTCH2NLA, NOTCH2NLB, NOTCH2NLR, and NOTCH2, along with uN2CpolyG and PolyGN2C-iso2. The PolyG repeat region is highlighted with dashed rectangles, and the epitope regions recognized by M38D5, M11F6, and 4C4 antibodies are marked with bold horizontal lines. (**E**) U2OS cells were transfected with the TV2-PolyG-Flag construct containing 64xGGC repeats. Immunofluorescence analysis performed at 48 hours post-transfection demonstrated colocalization of Flag-tagged PolyG(64×)N2C-iso2 protein with positive signals from both M38D5 and M11F6 monoclonal antibodies. **(F)** Immunoblots of lysates from U2OS cells transfected with TV2-PolyG-Flag constructs containing 14× or 64× GGC repeats, or vector control. Blots were probed with the newly generated PolyGN2C-iso2 monoclonal antibodies M38D5 and M11F6. Both antibodies detected high-molecular-weight PolyG(64×)N2C-iso2 species above 250 kDa.


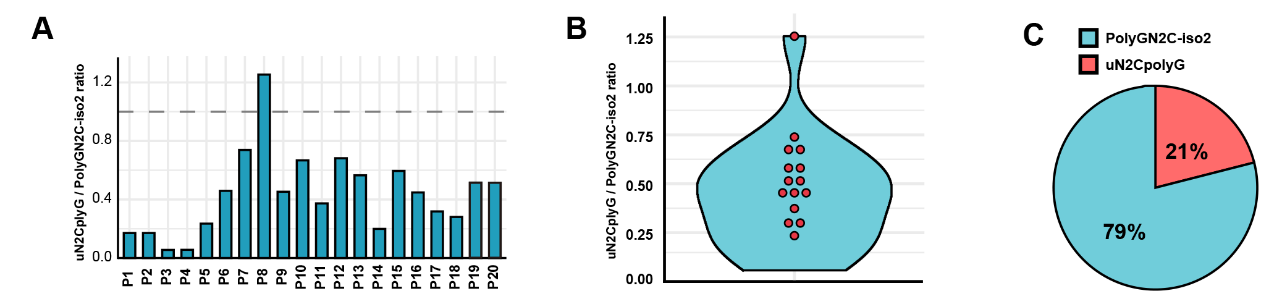


**Figure S3 Related to Figure 3**

**(A)** Ratio of Skyline-extracted total peak areas for the selected uN2CpolyG-associated surrogate peptide and the PolyGN2C-iso2-discriminating surrogate peptide in individual NIID patient samples. **(B)** Violin plot showing the distribution of uN2CpolyG/PolyGN2C-iso2 surrogate-peptide signal ratios across NIID patient samples. **(C)** Relative contribution of the selected uN2CpolyG- and PolyGN2C-iso2-associated surrogate peptides to the combined NOTCH2NLC-derived surrogate-peptide signal, based on summed Skyline-extracted total peak areas. PolyGN2C-iso2 accounted for approximately 79% and uN2CpolyG for approximately 21% of the combined surrogate-peptide signal.


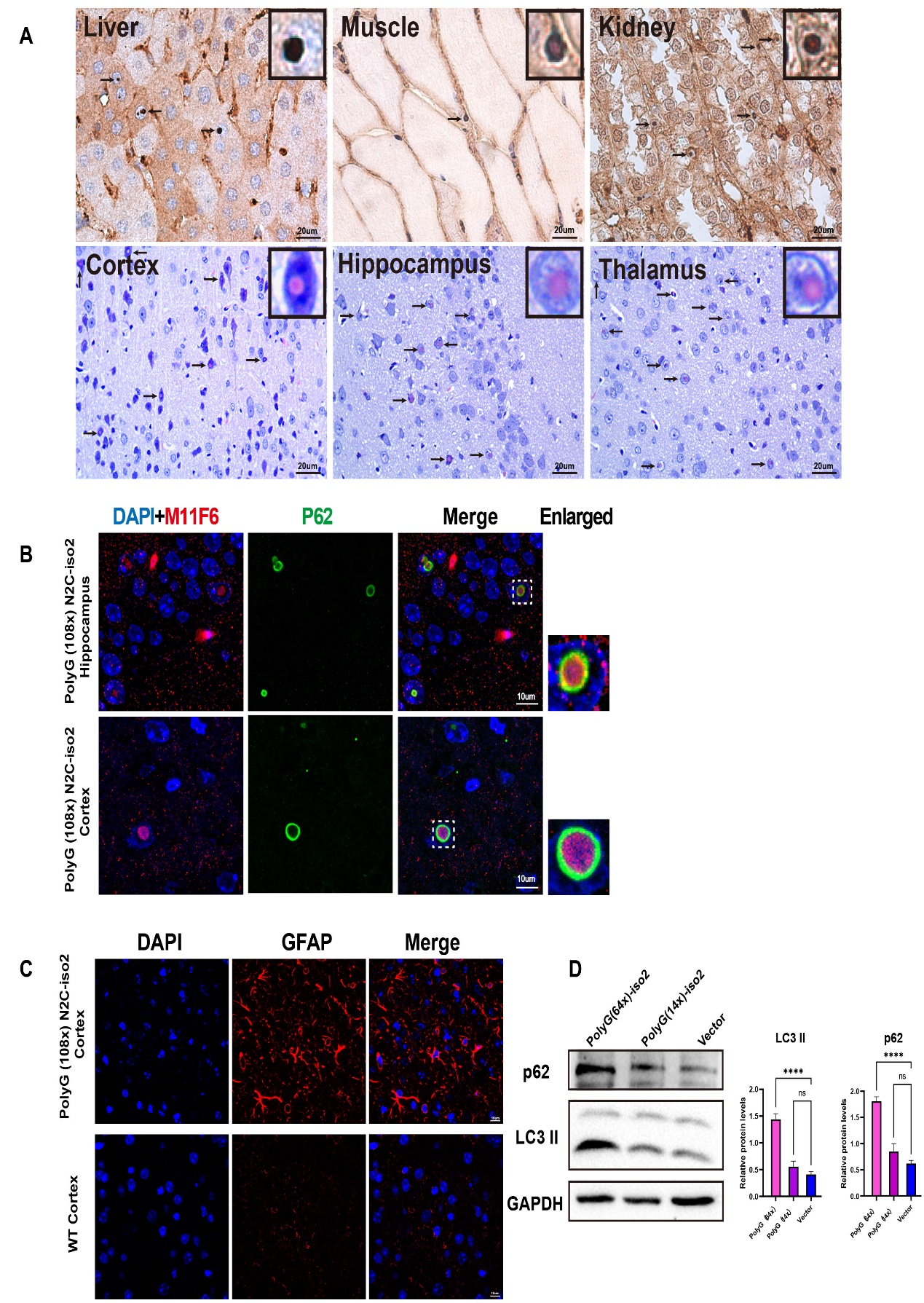


**Figure S4 Related to Figure 4**

**(A)** Representative immunohistochemistry (IHC) and HE staining images of various tissues from PolyG(108×)N2C-iso2 model mice. IHC staining of the liver, muscle, and kidney, with arrows indicating p62-positive nuclear inclusions (top). HE staining of the cerebral cortex, hippocampus, and thalamus, with black arrows highlighting eosinophilic nuclear inclusions in each brain region (bottom). **(B)** Representative immunofluorescence images of M11F6 and p62 double staining in the cerebral cortex and hippocampus of PolyG(108×)N2C-iso2 model mice. **(C)** Representative immunofluorescence images of GFAP staining in brain tissues from PolyG(108×)N2C-iso2 model mice and WT mice. **(D)** Representative Western blot analysis of p62 and LC3-II, key autophagy markers, in U2OS cells transfected with the TV2-PolyG-Flag construct (left). Quantification bar graph showing relative protein levels (right). Data represent the mean ± SEM from three independent biological replicates (*n*=3). Statistical analysis was performed using one-way ANOVA with Tukey's multiple comparisons test; *****P*<0.0001.


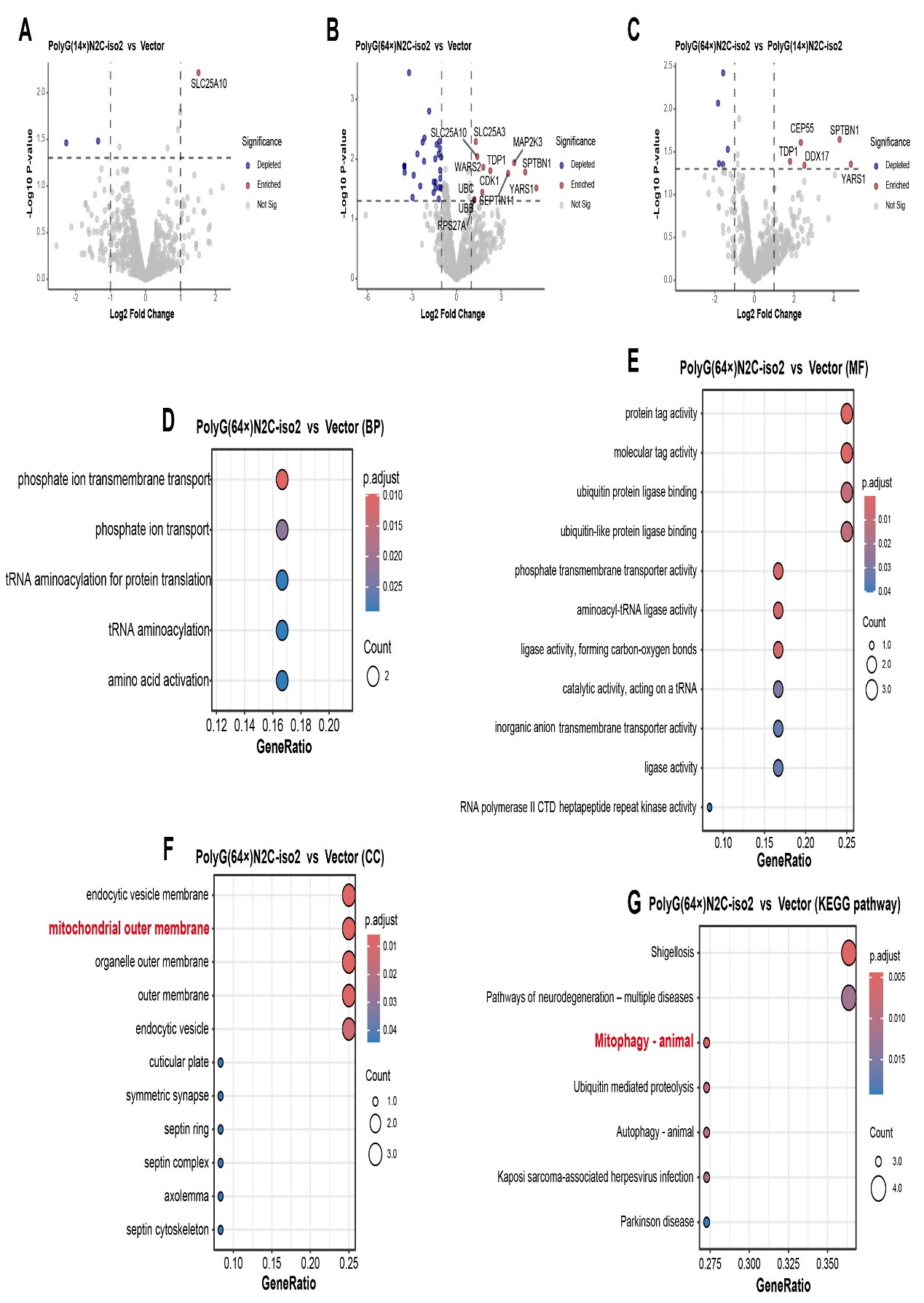


**Figure S5 Related to Figure 6**

FLAG-IP/MS was performed in U2OS cells transfected with empty vector, PolyG(14×)N2C-iso2-Flag, or PolyG(64×)N2C-iso2-Flag constructs, with three biological replicates per group. **(A-C)** Volcano plots showing differentially enriched proteins in the comparisons of PolyG(14×)N2C-iso2 versus vector **(A)**, PolyG(64×)N2C-iso2 versus vector **(B)**, and PolyG(64×)N2C-iso2 versus PolyG(14×)N2C-iso2 **(C)**. The x-axis represents log_2_fold change, and the y-axis represents -log_10_P value. Significantly enriched and depleted proteins are shown in red and blue, respectively, while non-significant proteins are shown in grey. Dashed lines indicate the cutoff thresholds. Candidate proteins are labeled. High-confidence candidate interactors were defined as proteins with >1 unique peptide, |log_2_FC| > 1, and P < 0.05. **(D-F)** Gene Ontology enrichment analysis of high-confidence proteins enriched in the PolyG(64×)N2C-iso2 pulldown compared with vector control. Representative enriched terms are shown for Biological Process **(D)**, Molecular Function **(E)**, and Cellular Component **(F)** categories. **(G)** KEGG pathway enrichment analysis of high-confidence proteins enriched in the PolyG(64×)N2C-iso2 pulldown compared with vector control.

**Supplementary Tables**

**Table S1. Clinical features of NIID cases and control individuals.**

| **Clinical information** | **NIID-01** | **NIID-02** | **NIID-03** | **NIID-04** | **NIID-05** | **NIID-06** | **Normal-01** |
| --- | --- | --- | --- | --- | --- | --- | --- |
| **Gender** | Male | Male | Male | Female | Female | Female | Male |
| **Family history** | Sporadic | Sporadic | Sporadic | Sporadic | Sporadic | Sporadic | **-** |
| **Survival status** | alive | alive | alive | alive | alive | alive | **-** |
| **Age at onset (years)** | 60 | 53 | 49 | 56 | 62 | 68 | **-** |
| **Disease duration (months)** | 36 | 51 | 48 | 61 | 108 | 35 | **-** |
| **Clinical classification** |  |  |  |  |  |  | **-** |
| Cognitive impairment-dominant type | **-** | **-** | **+** | **-** | **-** | **+** | **-** |
| Episodic neurogenic event-dominant type | **-** | **+** | **-** | **-** | **+** | **-** | **-** |
| Movement disorder-dominant type | **-** | **-** | **-** | **-** | **-** | **-** | **-** |
| Autonomic dysfunction-dominant type | **+** | **-** | **-** | **-** | **-** | **-** | **-** |
| Neuromuscular disease-dominant type | **-** | **-** | **-** | **+** | **-** | **-** | **-** |
| **Changes in brain MRI findings** |  |  | **-** |  |  |  | **-** |
| Prominent white matter abnormalities | **+** | **+** | **+** | **+** | **+** | **+** | **-** |
| Hyperintense signals in the corticomedullary junction on DWI | **+** | **+** | **+** | **+** | **+** | **+** | **-** |
| Prominent ventricular enlargement | **+** | **+** | **+** | **+** | **+** | **+** | **-** |
| **Skin biopsy** | **+** | **+** | **+** | **+** | **+** | **+** | **+** |
| **Muscle biopsy** | **-** | **-** | **-** | **+** | **+** | **+** | **-** |
| **GGC repeats in the *NOTCH2NLC* gene** | 85 | 102 | 109 | 118 | 93 | 110 | **-** |

**Table S2.** **Demographic and clinical characteristics for NIID patients and control cohorts selected for proteomic analysis.**

| **Case ID** | **Case Type** | **Sex** | **Age at onset (years)** | **Disease Duration (years)** | **Clinical Subtype#** | **GGC(n)** |
| --- | --- | --- | --- | --- | --- | --- |
| NIID-07 | Familial | Male | 60 | 5 | 4 | 113 |
| NIID-08 | Sporadic | Female | 64 | 4 | 1 | 102 |
| NIID-09 | Familial | Female | 40 | 14 | 4 | 122 |
| NIID-10 | Familial | Male | 47 | 14 | 4 | 129 |
| NIID-11 | Sporadic | Male | 49 | 3 | 1 | 131 |
| NIID-12 | Sporadic | Female | 42 | 11 | 2 | 115 |
| NIID-13 | Sporadic | Female | 45 | 2 | 1 | 124 |
| NIID-14 | Familial | Female | 46 | 30 | 3 | 116 |
| NIID-15 | Sporadic | Female | 64 | 3 | 1 | 96 |
| NIID-16 | Sporadic | Female | 56 | 1 | 2 | 118 |
| NIID-17 | Familial | Male | 54 | 10 | 2 | 114 |
| NIID-18 | Sporadic | Female | 55 | 2 | 1 | 123 |
| NIID-19 | Sporadic | Male | 71 | 1 | 4 | 105 |
| NIID-20 | Familial | Male | 48 | 20 | 3 | 128 |
| NIID-21 | Sporadic | Male | 51 | 9 | 1 | 93 |
| NIID-22 | Familial | Male | 56 | 2 | 1 | 115 |
| NIID-23 | Sporadic | Male | 61 | 5 | 3 | 153 |
| NIID-24 | Sporadic | Female | 35 | 27 | 3 | 180 |
| NIID-25 | Familial | Female | 58 | 1 | 2 | 128 |
| NIID-26 | Sporadic | Female | 61 | 6 | 5 | 149 |
| Normal-2 | - | Male | - | - | - | - |
| Normal-3 | - | Male | - | - | - | - |
| Normal-4 | - | Male | - | - | - | - |
| Normal-5 | - | Female | - | - | - | - |
| Normal-6 | - | Female | - | - | - | - |
| Normal-7 | - | Female | - | - | - | - |

#Notes on Clinical Subtype:1= Cognitive impairment-dominant type. 2= Episodic neurogenic event-dominant type. 3= Movement disorder-dominant type. 4= Autonomic dysfunction-dominant type. 5= Neuromuscular disease-dominant type.

**Table S3. Comparison of the PolyGN2C-iso2 interactome with previously reported uN2CpolyG interactomes.**

| **Gene** | **Accession** | **Liu et al. uN2CpolyG interactome** | **Boivin et al. uN2CpolyG interactome** | **Peptides** | **Unique** |
| --- | --- | --- | --- | --- | --- |
| **RACK1** | P63244 |  | shared | 1 | 1 |
| **CBX3** | Q13185 |  | shared | 6 | 5 |
| **FASN** | P49327 |  | shared | 1 | 1 |
| **SLC25A5** | P05141 | shared | shared | 17 | 5 |
| **DNAJA1** | P31689 |  | shared | 9 | 9 |
| **PRDX4** | Q13162 |  | shared | 1 | 1 |
| **EIF4A1** | P60842 | shared |  | 2 | 1 |
| **XRCC6** | P12956 | shared | shared | 4 | 4 |
| **XRCC5** | P13010 | shared | shared | 3 | 3 |
| **TUFM** | P49411 |  | shared | 14 | 14 |
| **DDB1** | Q16531 |  | shared | 1 | 1 |
| **MTHFD1** | P11586 |  | shared | 4 | 4 |
| **FBL** | P22087 | shared |  | 1 | 1 |
| **RPS6** | P62753 | shared |  | 5 | 5 |
| **PRKDC** | P78527 |  | shared | 11 | 11 |
| **IGKV2D-29** | A0A075B6S2 | shared |  | 2 | 2 |
| **RPL18A** | Q02543 | shared |  | 6 | 6 |
| **RARS1** | P54136 |  | shared | 9 | 9 |
| **SRSF11** | Q05519 | shared |  | 3 | 3 |
| **RPS24** | P62847 | shared |  | 4 | 4 |
| **HNRNPF** | P52597 | shared |  | 2 | 2 |
| **EEF2** | P13639 |  | shared | 4 | 3 |
| **RPL22** | P35268 | shared |  | 10 | 9 |
| **SF3A1** | Q15459 | shared |  | 18 | 18 |
| **PRMT5** | O14744 |  | shared | 3 | 3 |
| **H3C2** | P68431 |  | shared | 2 | 2 |
| **HNRNPM** | P52272 | shared |  | 2 | 2 |
| **SRSF2** | Q01130 | shared |  | 5 | 5 |
| **TUBB** | P07437 | shared |  | 31 | 2 |
| **ABCB1** | P08183 | shared |  | 1 | 1 |
| **RPL34** | P49207 | shared |  | 1 | 1 |
| **SNRPD2** | P62316 | shared |  | 4 | 4 |
| **CTPS1** | P17812 |  | shared | 5 | 5 |
| **FLNA** | P21333 |  | shared | 3 | 2 |
| **CAD** | P27708 |  | shared | 7 | 7 |
| **SF3B4** | Q15427 | shared |  | 1 | 1 |
| **IGHG2** | P01859 | shared |  | 3 | 2 |
| **YARS1** | P54577 |  |  | 9 | 9 |
| **SPTBN1** | Q01082 |  |  | 11 | 11 |
| **MAP2K3** | P46734 |  |  | 2 | 2 |
| **SEPTIN11** | Q9NVA2 |  |  | 5 | 2 |
| **TDP1** | Q9NUW8 |  |  | 2 | 2 |
| **WARS2** | Q9UGM6 |  |  | 2 | 2 |
| **CDK1** | P06493 |  |  | 5 | 5 |
| **SLC25A10** | Q9UBX3 |  |  | 3 | 3 |
| **SLC25A3** | Q00325 |  |  | 5 | 5 |
| **RPS27A** | P62979 |  |  | 3 | 3 |
| **UBB** | P0CG47 |  |  | 3 | 3 |
| **UBC** | P0CG48 |  |  | 3 | 3 |

**Caption:** Summary table comparing proteins detected in the PolyGN2C-iso2 FLAG-IP/MS dataset with previously reported uN2CpolyG-associated proteins from Liu et al. and Boivin et al. Proteins shown in red indicate proteins detected in the PolyG(64×)N2C-iso2 pulldown that were previously reported in uN2CpolyG interactomes; these shared proteins were not selected based on statistical enrichment in the present dataset. Proteins shown in black indicate high-confidence candidate interactors significantly enriched in the pathogenic PolyG(64×)N2C-iso2 pulldown compared with vector control (*p* < 0.05).

**Table S4. List of** **antibodies**

| **Items** | **Company** | **Cat#** | **Assay** | **Dilution/Final Concentration** |
| --- | --- | --- | --- | --- |
| rabbit anti-p62/SQSTM1 | Proteintech | 18420-1-AP | IF  IHC  WB | 1:800  1:800  1:5000 |
| rabbit or mouse anti-DYKDDDDK (Flag) | Proteintech | 20543-1-AP | IF | 1:1200 |
| rabbit anti-HA | Proteintech | 66008-4-Ig | IF | 1:1200 |
| monoclonal rabbit anti-4C4 | BGI Genomics Co., Ltd. | custom-made | IF | 1:800 |
| mouse anti-M38D5 | BGI Genomics Co., Ltd. | custom-made | IF | 1:400/  5.5 µg/mL |
| mouse anti-M11F6 | BGI Genomics Co., Ltd. | custom-made | IF | 1:400/  6.75 µg/mL |
| MFN1 | Proteintech | 13798 | WB | 1:2000 |
| MFN2 | Proteintech | 12186 | WB | 1:5000 |
| DRP1 | Proteintech | 12957 | WB | 1:2000 |
| LC3B | CST | 3868S | WB | 1:1000 |
| GAPDH | Abcam | ab8245 | WB | 1:5000 |

**Legends for movie S1**

This video captures a spontaneous seizure-like event in a PolyG(108×)N2C-iso2 mouse. The behavioral phenotype includes a combination of involuntary head nodding, unilateral forelimb clonus, generalized body tremor, and pronounced tail extension.
